# Differential gene expression and phenotypic variation across tissues between *Saccharum officinarum* and *Saccharum spontaneum*

**DOI:** 10.3389/fpls.2025.1696921

**Published:** 2025-10-31

**Authors:** Qin-Nan Wang, Sheng-Ren Sun, Jun-Lv Chen, Jing-Ru Zhou, Yuan-Xia Qin, Wei Zhang, Li-Yu Chen, San-Ji Gao

**Affiliations:** ^1^ National Engineering Research Center of Sugarcane, Fujian Agriculture and Forestry University, Fuzhou, Fujian, China; ^2^ Institute of Nanfan & Seed Industry, Guangdong Academy of Sciences, Guangzhou, Guangdong, China; ^3^ National Key Laboratory for Tropical Crop Breeding, Haikou, Hainan, China; ^4^ College of Agriculture, Fujian Agriculture and Forestry University, Fuzhou, China

**Keywords:** differentially expressed genes, phenotypic variation, *Saccharum* species, transcriptome analysis, tissue-specific expression

## Abstract

Modern sugarcane cultivars (*Saccharum* spp.), predominantly derived from *Saccharum officinarum* and *Saccharum* sp*ontaneum*, exhibit divergent traits in sugar content, yield, and stress tolerance. However, little is known about the spatial transcriptional regulation pattern of the differentially expressed genes (DEGs) among tissues in the two *Saccharum* species. This study aimed to investigate these genes according to pathways in root, stem, leaf, and flower tissues using comparative transcriptome analysis. A broad range of DEGs, ranging from 24,469 to 34,198, were identified in four tissues from Badila (*S. officinarum*) and Ledong2 (*S.* sp*ontaneum*) clones. Thirty-one DEGs involved in abscisic acid (ABA) biosynthesis were identified in the root, suggesting differences in ABA content within root tissues between the two clones. In stem tissues, a significant number of upregulated DEGs were associated with cell growth and division, alongside plant-type cell wall organization, while abundant downregulated DEGs were linked to stress-response processes, possibly contributing to heterogeneity in stem morphology and stress responses. In leaf tissues, DEGs related to photosynthesis and photorespiration pathways likely influenced the variation in plant biomass and sucrose content between Badila and Ledong2. Key DEGs, including *LHY*, *PRR7*, and *GI*, associated with circadian rhythms and the photoperiodic pathway, were identified in flower tissues, providing evidence to explain the discrepancy in flowering time between the two clones. Collectively, the differential regulation of genes across four tissues may contribute to illustrating the divergence of agronomic traits and stress responses in both *Saccharum* species, offering a valuable foundation for the genetic improvement of sugarcane cultivation and stress resilience.

## 1 Introduction

Sugarcane (*Saccharum* spp.), a key C_4_ plant in the Poaceae family, plays a vital role in the global production of sugar and biofuel, accounting for approximately 80% sugar and 40% ethanol production, respectively (Aono et al., 2021). The cultivation and production statistics from 2023 underscore its significance, with a recorded 202.58 million tons of sugar produced from 2.70 million hectares globally. China stands out as the third-largest sugar-producing country, cultivating approximately 0.130 million hectares and producing 10.46 million tons of sugar (https://www.fao.org/faostat/en/#data/QCL/visualize). Modern sugarcane cultivars possess a highly complex genome and polyploidy (2n = 100–130) via interspecific hybridization between *Saccharum officinarum* (80% chromosomes) and *Saccharum* sp*ontaneum* (10%–15% chromosomes) and 5%–10% recombinant chromosomes (D’Hont, 2005; Wang et al., 2022a). The two *Saccharum* species mainly contributed to the genetic background of modern sugarcane cultivars (Jiang et al., 2023). The high sugar content of modern sugarcane cultivars originated from *S. officinarum*, while hardiness, stress tolerance, and ratooning capacity originated from *S.* sp*ontaneum* through the nobilization breeding program (Jiang et al., 2023; Thirugnanasambandam et al., 2023). Increasing sugar content, photosynthetic efficiency, biomass accumulation, and tolerance to stress is an important project for modern sugarcane breeding programs, but the current progress is extremely slow due to the homogeneity background of hybrid parents (Calderan-Rodrigues et al., 2021).

Transcriptome analysis by high-throughput RNA sequencing (RNA-seq) has become a crucial tool for understanding the gene regulatory networks and discovering novel genes involved in the regulation of plant growth and development, as well as defense response to stressors (Ali et al., 2019; Yang et al., 2021). In sugarcane, transcriptomic data revealed that the differentially expressed genes (DEGs) associated with photosynthesis, phytohormone metabolism, and cell wall synthesis are involved in gibberellin (GA)-mediated sugarcane internode growth (Chen et al., 2020). The DEGs linked to sucrose metabolic pathways present in stalks were revealed using comparative transcriptomics during the sugar accumulation stage (Khan et al., 2021). A pan-transcriptome analysis on sugarcane revealed that a crucial gene *ScHCT* encoding an enzyme for lignin synthesis is involved in the regulation of lignin and sugar contents (Chen et al., 2025). Divergences of the photosynthesis pathway between *S. officinarum* and *S.* sp*ontaneum* were demonstrated using transcriptomic dynamics analysis (Jiang et al., 2023). In addition, transcriptome analysis is widely used to elucidate some key molecular mechanisms involved in tolerance and adaptability to stressors. For example, transcriptomic responses of genes associated with abscisic acid (ABA) metabolism and signal transduction were upregulated in *S.* sp*ontaneum* root tips during early drought stress (Wu et al., 2022). A spatiotemporal transcriptome analysis on both aerial stems and rhizomes in the clone Bainianzhe demonstrated that some genes related to ABA and GA synthesis were highly expressed in rhizomes and that photosynthesis-related genes were enriched in the apical segments of rhizomes (Li et al., 2025).

It is well known that the transition from vegetative to reproductive growth in flowering plants such as sugarcane plays a crucial role in the growth and development stages controlling the flowering. Transcriptome analysis revealed that photoperiodic response and flowering time-related genes play critical roles during flowering induction in sugarcane under controlled photoperiodic conditions (Manechini et al., 2021). Moreover, the circadian, photoperiod, and GA pathways were responsive in floral development in sugarcane (Pavani et al., 2023). Comparison of transcriptome and weighted gene correlation network analysis (WGCNA) between male-sterile *S. officinarum* (LA Purple) and fertile *S.* sp*ontaneum* (SES208) showed that numerous genes or alleles and some phytohormone signaling pathways (GA, auxin, and jasmonic acid) are associated with the male sterility in sugarcane (Song et al., 2024).

The genetic improvement of sugarcane in mainland China commenced with conventional hybrid breeding in the early 1950s (Qi et al., 2020). Numerous modern sugarcane cultivars originated from Yacheng (YC) serial clones that were generated from two ancient parents of Badila (*S. officinarum*) and Ledong2 (*S.* sp*ontaneum*) in China (Deng et al., 2004; Chen et al., 2010). For instance, two main Chinese sugarcane cultivars, GT42 and GT44, are the third-generation hybrid offspring of YC58-47 (Badila × Ledong2). Therefore, in this study, we characterized and analyzed the transcriptional changes of key genes participating in ABA response in roots, photosynthetic efficiency in leaves, stem growth and development, and flowering in Badila and Ledong2 based on comparative transcriptome analysis and reverse transcription–quantitative polymerase chain reaction (RT-qPCR) assays. Our findings offer a novel understanding of the molecular mechanisms underlying sugarcane growth and development and provide candidate genes for the genetic improvement of sugarcane.

## 2 Material and methods

### 2.1 Plant material and RNA-sequencing

Two clones, Ledong2 and Badila, were planted in Sugarcane Germplasm Nursery (E 109°09′59″, N 18°21′27″) in Sanya, Hainan Province, China. Four tissue samples (the top visible dewlap leaf blades without midrib, middle stem, root, and flower) were collected from healthy plants (12 months old) of both clones. Ledong2_Leaf, Ledong2_Stem, Ledong2_Root, and Ledong2_Flower were from various tissues in Ledong2; Badila_Leaf, Badila_Stem, Badila_Root, and Badila_Flower were from various tissues in Badila. Three biological samples for each tissue were collected from different plants. Thus, a total of 24 samples were used for Illumina RNA-seq. RNA isolation was performed using the Arcturus PicoPure RNA isolation Kit (Applied Biosystems, Foster City, CA, USA). The cDNA library preparations were sequenced on an Illumina HiSeq 6000 platform at Berry Genomics Corporation (Beijing, China), and 150-bp paired-end sequencing reads were generated. The sequence file has been uploaded to the National Center for Biotechnology Information (NCBI) Sequence Read Archive (SRA) under the accession number PRJNA873207.

### 2.2 Transcriptome assembly and data analysis

The sequencing quality of raw reads was evaluated using Fastp (fastp: an ultra-fast all-in-one FASTQ preprocessor) to remove low-quality reads with several criteria: 1) adapter and PCR primer sequences were removed, 2) leading and trailing bases with low quality were removed, 3) bases with average quality below 20 in a 5-base sliding window were dropped, and 4) final reads shorter than 50 bp were dropped. High-quality paired-end reads were then aligned to the sugarcane reference genome (Garsmeur et al., 2018; Zhang et al., 2018) with hisat2 (Kim et al., 2015). With properly aligned reads retained, each gene with aligned count was quantified using HT-Seq (Anders et al., 2015). Gene expression levels were quantified using transcripts per million (TPM) to standardize for sequencing depth differences between samples. DEGs between comparisons were identified using log_2_Fold Change (FC) >1 or <−1, with a *p*-value<0.05. The *p*-values were adjusted using the false discovery rate (FDR) method to correct for multiple hypothesis testing (Robinson et al., 2010). Gene ontology (GO) enrichment analysis and Kyoto Encyclopedia of Genes and Genomes (KEGG) enrichment were performed using clusterProfiler (Yu et al., 2012) to understand the functional roles of those DEGs. These GO and KEGG terms with a *p*-value<0.05 were regarded as significantly enriched. Heatmaps were drawn using “pheatmap” (Kim et al., 2015; Kolde and Kolde, 2015) with genes involved in a specific GO or KEGG term. To understand the biological processes involved in a specific pathway, MAPMAN (Thimm et al., 2004) was also used to identify the functional categories of DEGs.

### 2.3 Test of photosynthetic parameters in sugarcane leaves

A portable chlorophyll fluorometer PAM-2500 (Walz, Effeltrich, Germany) was used to measure the chlorophyll fluorescence induction kinetic curve and fast light response curve in sugarcane leaves, as previously described by Loggini et al. (1999). After the leaf blade was adapted to dark conditions for 30 min using leaf clips, it was initially exposed to the modulated measuring beam of far-red light (LED source with a typical peak at wavelength 735 nm). The effective photochemical quantum yield of photosystem II [Y(II)], half-saturation constant (IK), non-photochemical quenching (NPQ), and electron transport rate (ETR) were measured under weak modulated red light with 1.6-s pulses of saturating light (Maxwell and Johnson, 2000). The measurements were recorded with three replicates of each time point (7:00, 9:00, 11:00, 13:00, 15:00, 17:00, 19:00, and 21:00). Eight biological replications were used in each clone. The experiments were performed using two independent tests.

### 2.4 Determination of ABA content

The roots of sugarcane (approximately 0.15 g) were weighed, finely ground with liquid nitrogen, and then added with 1.5 mL of cooling phosphate-buffered saline (PBS) solution for extraction for 10 min at 4°C. Fifty microliters of supernatant was collected after centrifugation at 2,500 rpm for 10 min. The ABA content of each sample was determined in accordance with the instructions of the Plant Abscisic acid (ABA) ELISA Kit (Jiancheng Bioengineering Co., Ltd., Nanjing, China). Briefly, the lyophilized powder of the ABA reference standard was diluted in 7.5–120 ng/mL solutions to generate the calibration standard curve. The competitive ELISA procedure involved sequentially adding 50 µL of standard or sample and 50 µL of horseradish peroxidase (HRP)-conjugated ABA to microplates coated with anti-ABA antibody and then incubating for 1 h at 37°C in the dark. After this competitive binding step, 100 µL of substrate was added, and the plate was incubated for 20 min at 37°C in the dark. The reaction was stopped using 50 µL of stop solution. The absorbance value of ABA was quantified using the Synergy™ H1 Hybrid Multi-Mode Reader (BioTek, Winooski, VT, USA) at 450 nm. The above experiments were carried out on six biological samples for each clone.

### 2.5 Determination of stem internode and pollen germination rate

The micrometer was used to measure the length and width of the stem internode. A total of 30 stalks were randomly separated into six groups for determination in each clone. To explore the divergence of pollen development between Badila and Ledong2, fresh pollen collected from the flowers was scattered on a pollen germination medium that contained 15 g/L agar, 50 g/L sucrose, and 100 mg/L boric acid. Pollen on the medium was incubated for 3 h at 25°C. After incubation, the percentages of germinated pollen (pollen germination rate) were calculated. Germination of pollen rate was defined as the stage when the emerging pollen tube elongated to twice the diameter of the pollen grain. The mean percentage of pollen germination rate was calculated by averaging the percentages from three glass-slide samples. The pollen was collected at 8:30 am. Three biological replications were carried out for each clone.

### 2.6 Reverse transcription–quantitative PCR analysis

The cDNA was prepared in 500-ng RNA samples using PrimeScript RT Master Mix (Takara, Dalian, China). The qPCR was performed using LightCycler^®^ SYBR Green I Master (Roche Diagnostics, Mannheim, Germany) on the QuantStudio 5 Real-Time PCR system (Applied Biosystems, Foster City, CA, USA). Each reaction was performed in a final volume of 20 µL containing 10 µL of SYBR Green I Master mix, 1 µL of 10 µM forward primer, 1 µL of 10 µM reverse primer, 2.0 µL of the cDNA sample, and distilled water to the final volume. The qPCR cycling was performed at 95°C for 5 min, 40 cycles at 95°C for 10 s, 60°C for 20 s, and 72°C for 20 s. The glyceraldehyde 3-phosphate dehydrogenase (*GAPDH*) gene was used as a reference gene to normalize the data. The quantification method (2^−ΔΔCt^) was used to determine the differences in gene expression. Each sample was carried out with three biological and three technical replicates. The primer pairs used in this study are listed in **Supplementary Table S1**.

### 2.7 Statistical analysis

Significance tests were evaluated using one-way ANOVA, and the statistical significance of the means (*p*<0.05) was determined using t-tests between groups. All statistical analyses were performed using the SPSS 22.0 software.

## 3 Results

### 3.1 Assembly and annotation of transcriptomes from two *Saccharum* species

**Figure 1:**
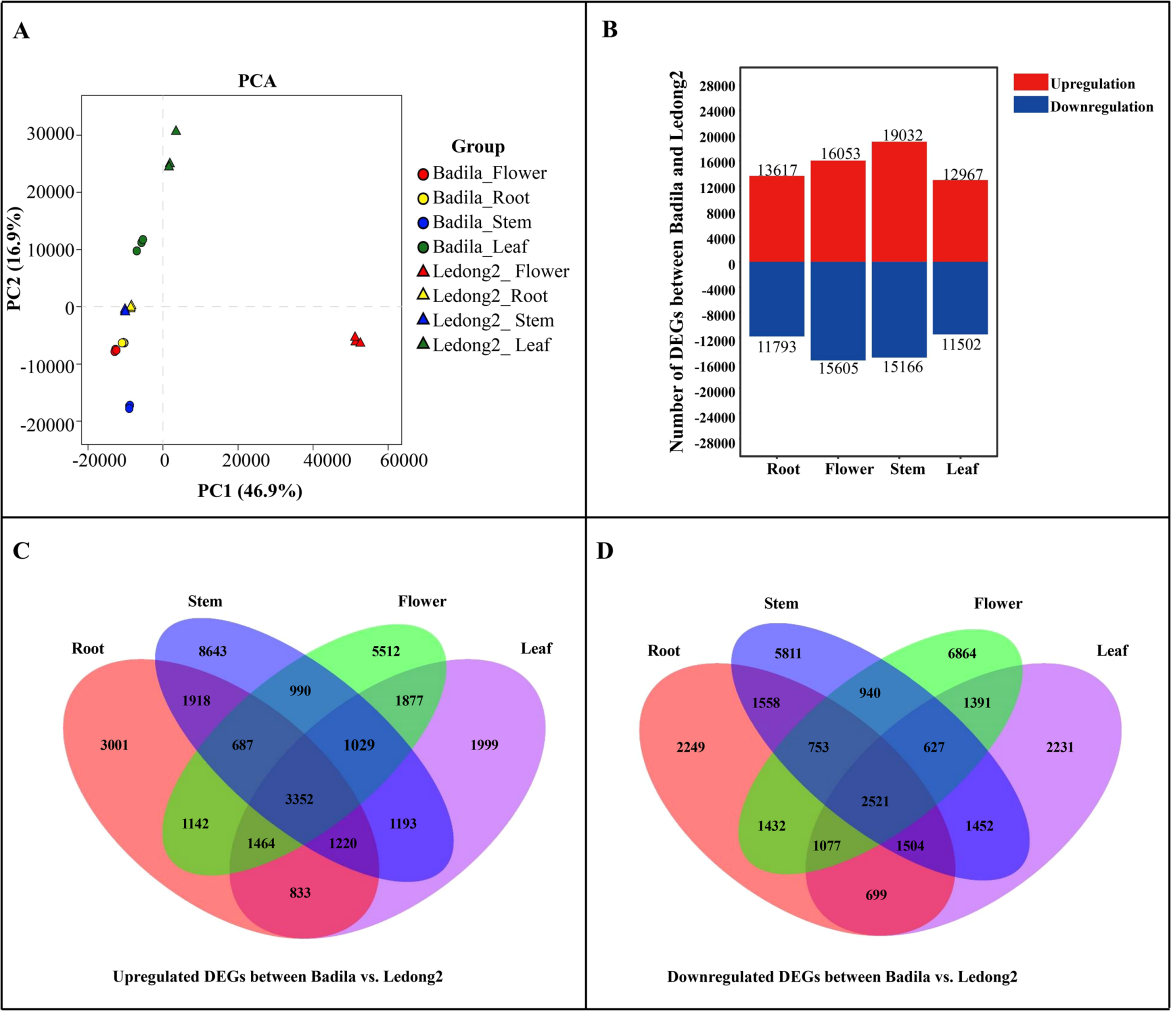
Principal component analysis (PCA) and comparative analysis of differentially expressed genes (DEGs) between Badila and Ledong2. **(A)** Principal component analysis. **(B)** DEG numbers identified in four tissues. (**C**, **D**) Upregulated and downregulated DEGs between Badila and Ledong2, respectively.

To systematically analyze the gene expression differences between Badila and Ledong2, RNA-seq libraries were constructed using four distinct tissue types: root, leaf, stem, and flower. Among 24 libraries, a substantial number of paired-end reads were obtained, ranging from 72.81 to 131.77 million bp, with more than 97.28% of clean reads being selected for subsequent analysis. After mapping these reads to the genome of *S.* sp*ontaneum* clone AP85-441, 35.64 to 64.57 million high-quality reads were retained for expression profiling (**Supplementary Table S2**). The transcripts from 24 libraries in the assembled transcriptome were quantified and normalized in terms of TPM to account for sequencing depth differences between samples. All the transcript expression profiles from different samples had a similar variation. For example, the median log_2_(TPM) values across all tissues spanned from 0.47 to 1.10, indicating a relatively consistent expression level across different tissues (**Supplementary Figure S1**). Principal component analysis (PCA) revealed that these genes associated with the first two principal components accounted for 63.8% of all the total variance, and the samples from the same tissue exhibited substantial repeatability (**Figure 1A**). Additionally, Pearson’s correlation analysis indicated a strong replication among our samples (**Supplementary Figure S2**).

### 3.2 Identification and functional annotation of DEGs between two *Saccharum* species

A total of 25,410 (13,617 upregulation and 11,793 downregulation), 24,469 (12,967 upregulation and 11,502 downregulation), 34,198 (19,032 upregulation and 15,166 downregulation), and 31,658 DEGs (16,053 upregulation and 15,605 downregulation) were identified in root, leaf, stem, and flower tissues in both clones Badila and Ledong2, respectively (**Figure 1B**). Of these upregulated DEGs, 3,001, 1,999, 8,643, and 5,512 genes were observed in root, leaf, stem, and flower tissues, respectively (**Figure 1C**), while among these downregulated DEGs, 2,249, 2,231, 5,811, and 6,864 genes were found in four tissues, respectively (**Figure 1D**). A total of 2,521 and 3,352 genes showed higher expression levels in Badila and Ledong2, respectively.

To further understand the biological function of these DEGs, GO enrichment analysis was performed for each tissue comparison of Badila and Ledong2. In the root tissue, the largest number of upregulated DEGs was enriched in response to ABA (353 genes), extracellular matrix (31 genes), and monooxygenase activity (123 genes) in the categories of biological process (BP), cellular component (CC), and molecular function (MF), respectively (**Supplementary Figure S3**). The maximum number of downregulated DEGs—pyruvate biosynthetic process (42 genes), plant-type cell wall (161 genes), and l-aspartate:2-oxoglutarate aminotransferase activity (10 genes)—were enriched in three categories (**Supplementary Figure S3**). In the stem tissues of the upregulated DEGs, the first top enrichment categories in BP, CC, and MF were microtubule-based movement (165 genes), kinesin complex (143 genes), and microtubule motor activity (143 genes), respectively (**Supplementary Figure S4**). For the downregulated DEGs, the first top enrichment categories in BP, CC, and MF were response to chitin (113 genes), peroxisome (168 genes), and protein serine/threonine/tyrosine kinase activity (48 genes), respectively (**Supplementary Figure S4**). In the leaf tissue, the most enriched upregulated DEGs were multicellular organismal homeostasis (18 genes), chloroplast stroma (469 genes), and glutathione binding (27 genes), while the most enriched downregulated DEGs were lipid oxidation (31 genes), plasmodesma (419 genes), and 4-coumarate-CoA ligase activity (15 genes) in the categories of BP, CC, and MF, respectively (**Supplementary Figure S5**). In the flower tissue, the most enriched upregulated DEGs were response to ABA (524 genes), chloroplast thylakoid membrane (294 genes), and calcium ion binding (93 genes), while the most enriched downregulated DEGs were microtubule-based movement (135 genes), kinesin complex (116 genes), and microtubule motor activity (116 genes) in the categories of BP, CC, and MF, respectively (**Supplementary Figure S6**).

Additionally, KEGG pathway analysis revealed the most enriched pathways for each tissue (**Supplementary Figure S7**): drug metabolism–other enzymes (115 genes) for upregulated DEGs and glycolysis/gluconeogenesis (139 genes) for downregulated DEGs in root tissues, DNA replication (132 genes) for upregulated DEGs and phenylalanine metabolism (71 genes) for downregulated DEGs in stem tissues, apelin signaling pathway (81 genes) for upregulated DEGs and phenylalanine metabolism (56 genes) for downregulated DEGs in leaf tissues, and photosynthesis proteins (101 genes) for upregulated DEGs and systemic lupus erythematosus (86 genes) for downregulated DEGs in flower tissues. Notably, the photosystem pathways (ko00194 and ko00195) were enriched in all tissues except for the root, while the DNA replication pathway (ko03030) was enriched in all tissues except for the leaf. The top 10 KEGG pathways in root, stem, leaf, and flower tissues from Badila and Ledong2 are shown in **Supplementary Table S3**.

### 3.3 Expression analysis of DEGs related to ABA synthesis and signaling pathways in root

**Figure 2:**
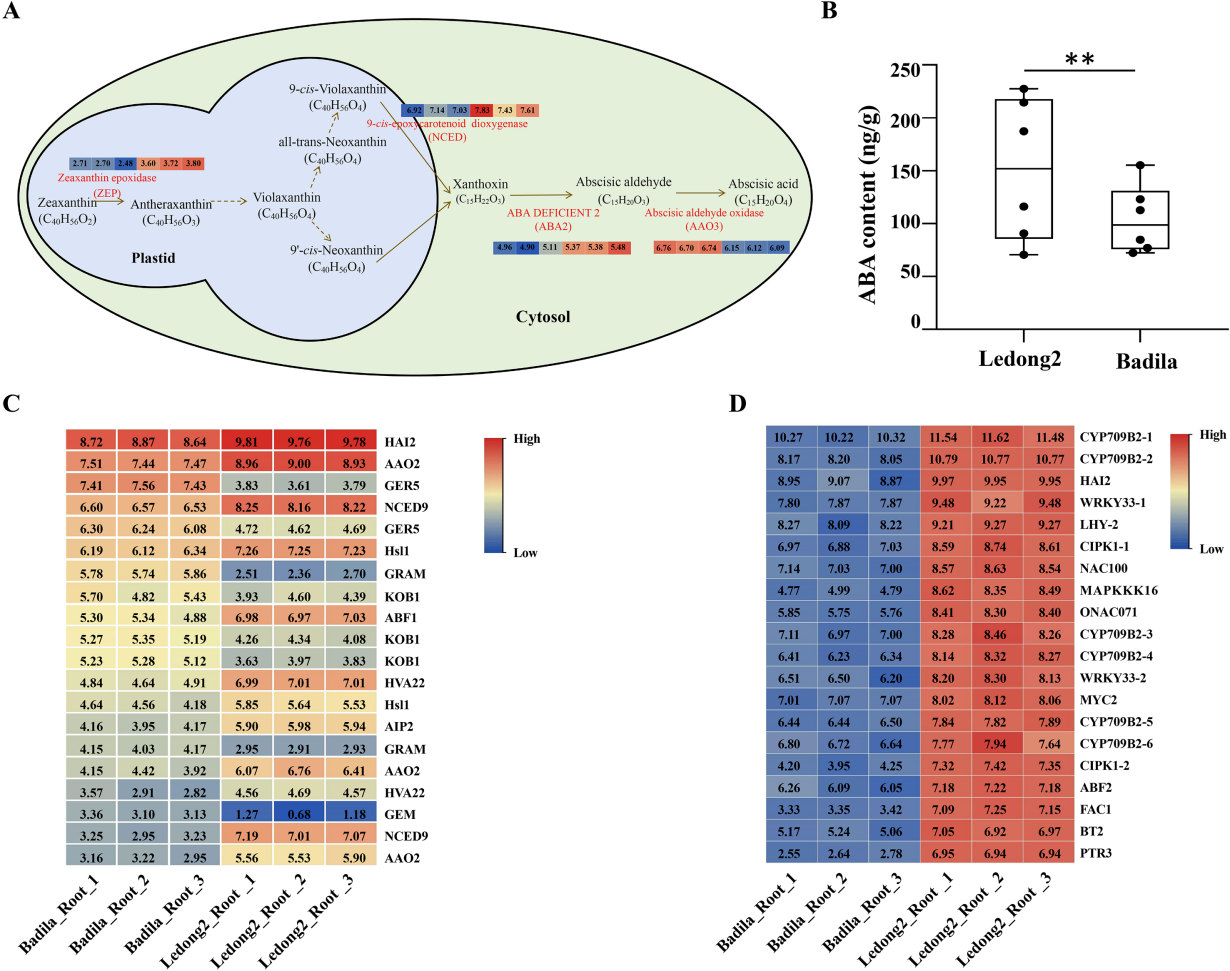
Transcript expression levels of differentially expressed genes (DEGs) involved in the abscisic acid (ABA) synthesis and the difference in ABA contents between Badila and Ledong2. **(A)** A sketch of ABA synthesis pathway. **(B)** ABA contents in roots (** *p*<0.01). **(C, D)** Transcript levels (log_2_TPM) of the representative DEGs involved in the ABA signal pathway and stress response, as well as development process, respectively.

The classical phytohormone ABA involves plant root meristem morphogenesis. In a comparative analysis between Badila and Ledong2, a total of 353 DEGs responsive to ABA (GO:0009737) were upregulated in the root of Badila (**Supplementary Figure S3** and **Supplementary Table S4**). Of them, 31 DEGs participated in the biosynthesis of ABA (**Figure 2A**), including the first limiting enzyme zeaxanthin epoxidase encoding gene *ScZEP* (three alleles), the key enzyme 9-*cis*-epoxycarotenoid dioxygenase encoding gene *ScNCED9* (seven alleles), *ScNCED4* (two alleles), *ScNCED3* (one allele), the ABA deficient 2 encoding gene *ScABA2* (three alleles), and the last key enzyme abscisic-aldehyde oxidase encoding gene *ScAAO3* (15 alleles). The majority of these genes were also upregulated in the root of Ledong2 (**Figure 2A**). ABA accumulation was significantly higher with a 1.4-fold increase in Ledong2 compared with Badila (**Figure 2B**), indicating that the elevated ABA level in Ledong2 is an important feature. In addition, transcript expression levels of genes related to the ABA signaling pathway are depicted in **Figure 2C**. The expression levels of these key genes, such as *ScNCED9*, *ABRE-binding transcription factor 1* (*ScABF1*), and *ScAAO2*, were upregulated in Ledong2. The transcript levels of 20 representative DEGs associated with stress responses and development processes are shown in **Figure 2D**. Overall, these genes exhibited higher transcript expression levels in Ledong2 compared with Badila. For instance, the transcript level (log_2_FC = 7.19) of the ABA regulator *ScABF2* in Ledong2 increased by 17.3% to the level (log_2_FC = 6.13) in Badila.

### 3.4 Expression analysis of DEGs related to cell elongation and differentiation in stem

**Figure 3:**
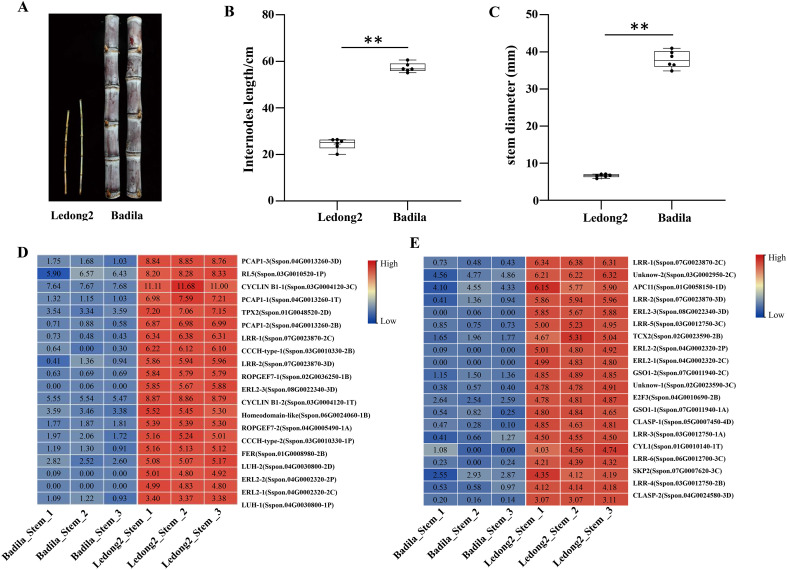
Morphological divergence and expression analysis of differentially expressed genes (DEGs) related to cell elongation and expansion in internodes between Badila and Ledong2. **(A–C)** Distinct morphological phenotype, internode length, and stalk diameter, respectively. (**D**, **E**) Transcript levels (log_2_FC) of the regulation of cell growth and cell division, respectively. ** *p*<0.01.

Two clones (Ledong2 and Badila) from different *Saccharum* species exhibited significant divergences of morphological traits such as internode length and stalk diameter. The average internode length was 24.47 cm in Ledong2 and 57.32 cm in Badila, while the average stem diameters were 6.63 mm in Ledong2 and 37.93 mm in Badila (**Figures 3A–C**). To further explore the possible mechanism for the regulation of cell elongation and expansion between the two clones, we examined the biological functions of those DEGs between Badila and Ledong2. A total of 19,032 upregulated DEGs were found to be associated with cell development and DNA metabolic processes, encompassing the regulation of cell growth (GO:0001558), regulation of cell division (GO:0051302), and plant-type cell wall organization (GO:0009664). Conversely, 15,166 downregulated DEGs were enriched in stress-response processes, including GO:0009611 and GO:1901002, indicating a potential difference in stress tolerance between the two clones (**Supplementary Figure S4** and **Supplementary Table S5**).

Among these DEGs, 72.3% were downregulated in the stem of Badila compared with Ledong2. For instance, the expression levels of DEGs involved in the regulation of cell growth and division were significantly higher in Ledong2, ranging from 290.0-fold (*Sspon.08G0022340-3D*) to 1.3-fold (*Sspon.03G0010520-1P* and *Sspon.03G0002950-2C*) than in Badila (**Figures 3D,E**). Notably, the expression levels of three *ERECTA-like2* genes (*ERL2-1/2/3*) ranged from 5.67 (log_2_FC) to 5.88 (log_2_FC) in Ledong2, whereas these genes exhibited negligible expression in Badila.

### 3.5 Expression analysis of DEGs related to photosynthetic efficiency in leaf

The results of GO enrichment analysis revealed that 12,967 genes, which were significantly highly expressed in Ledong2, were associated with photosynthetic electron transport in photosystem I (GO:0009773) and photosynthetic electron transport chain (GO:0009767) (**Supplementary Figure S5** and **Supplementary Table S6**). This suggests potential variation in photosynthesis efficiency between Badila and Ledong2.

**Figure 4:**
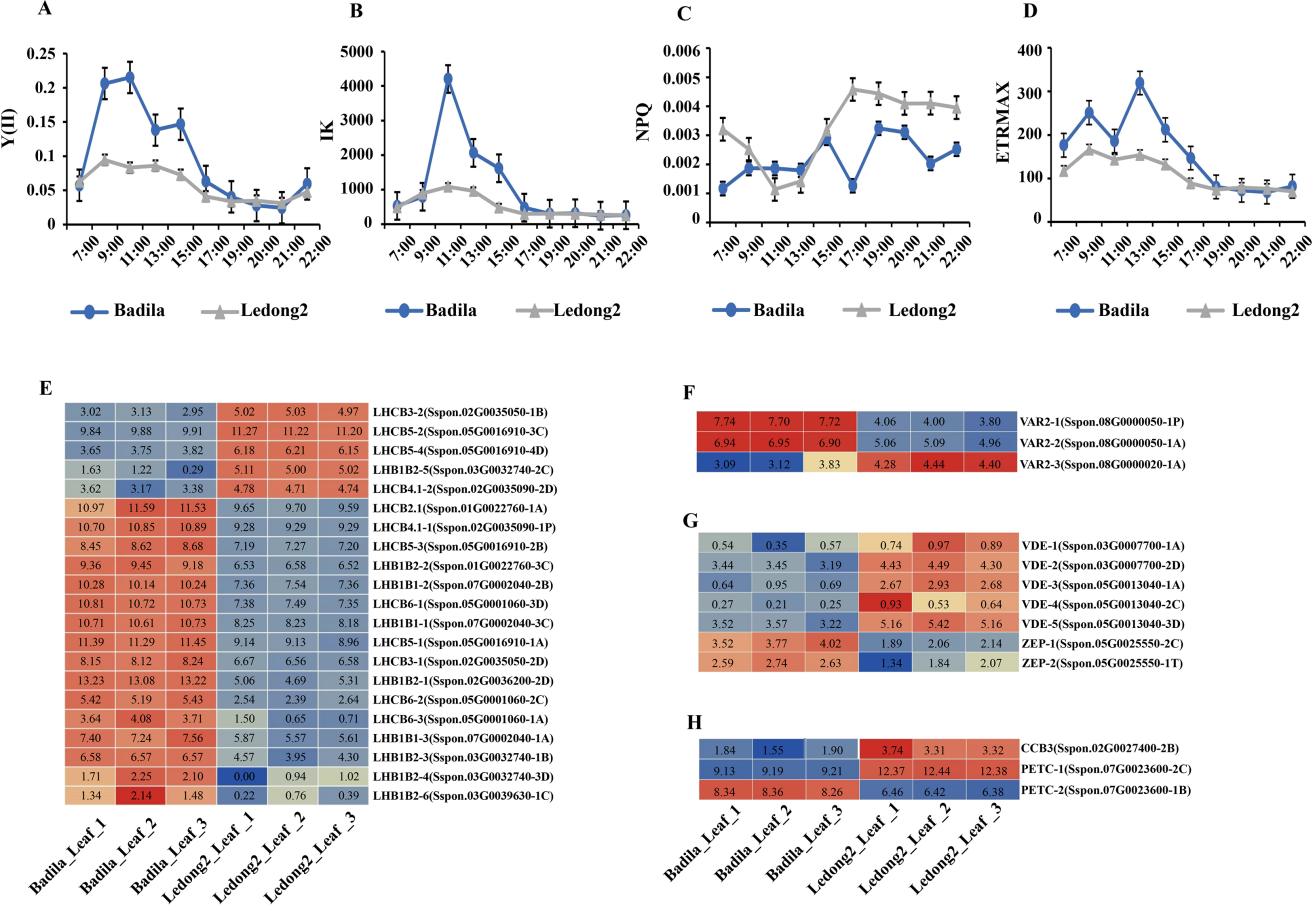
Photosynthetic parameters and transcript levels (log_2_FC) of differentially expressed genes (DEGs) related to photosynthetic and carbon fixation efficiency between Badila and Ledong2. **(A)** Effective photochemical quantum yield of photosystem II [Y(II)]. **(B)** Half-saturation constant (IK). **(C)** Non-photochemical quenching (NPQ). **(D)** Electron transport rate (ETR). **(E)** Photosystem II (PS II). **(F)** Photoinhibition. **(G)** Xanthophyll cycle. **(H)** Electron transport rate (ETR).

To assess the light tolerance and capture activity in sugarcane leaves of the two clones, physiological and biochemical parameters, including Y(II), IK, and NPQ, were measured. The light tolerance profiles for Y(II) and IK demonstrated a similar trend in both clones throughout the time flow from 7:00 am to 10:00 pm. However, Badila showed greater light tolerance than Ledong2 from 9:00 am to 3:00 pm (**Figures 4A,B**). The NPQ data indicated that Badila possessed a superior light capture ability and reduced light energy dissipation in photosystem II (PS II) yield during 7:00–9:00 am and 5:00–9:00 pm compared with Ledong2 (**Figure 4C**). Additionally, the ETR values showed that Badila possessed higher efficiency in carbon fixation through PS II from 7:00 am to 7:00 pm, particularly at 1:00 pm, compared with Ledong2 (**Figure 4D**).

The expression levels of 21 DEGs related to PS II light-harvesting complex were detected in both Badila and Ledong2. The majority of these DEGs (16/21) exhibited higher expression levels in Badila compared with Ledong2 (**Figure 4E**). Notably, the *VARIEGATED 2* (*VAR2*) genes that are involved in the repair of PS II following damage incurred during photoinhibition were significantly differently expressed between the clones. Two genes, *VAR2-1/2*, were significantly upregulated in Badila, whereas the *VAR2–3* gene was significantly downregulated (**Figure 4F**). In terms of the *cis*-xanthophyll cycle regulation, two genes encoding zeaxanthin epoxidase (*ScZEP*) were upregulated in Badila, while five genes related to violaxanthin de-epoxidase (*ScVDE*) were downregulated. Conversely, these seven genes exhibited opposite expression profiles in Ledong2 (**Figure 4G**). Additionally, two photosynthetic electron transport chain genes (*ScPETC*), which encode the Rieske FeS center of the cytochrome b6f complex and facilitate electron transport at saturating light intensities and carbon fixation, displayed varying expression levels. *PETC-1* (*Sspon.07G0023600-2C*) showed a higher expression level in Ledong2, whereas *PETC-2* (*Sspon.07G0023600-1B*) showed a higher expression level in Badila. The *ScCCB3* gene exhibited a similar expression pattern to *PETC-1* (**Figure 4H**). Together, these data revealed higher photosynthetic and carbon fixation efficiency, particularly at low light conditions in Badila compared with Ledong2.

### 3.6 Expression analysis of flowering-related genes in flower

**Figure 5:**
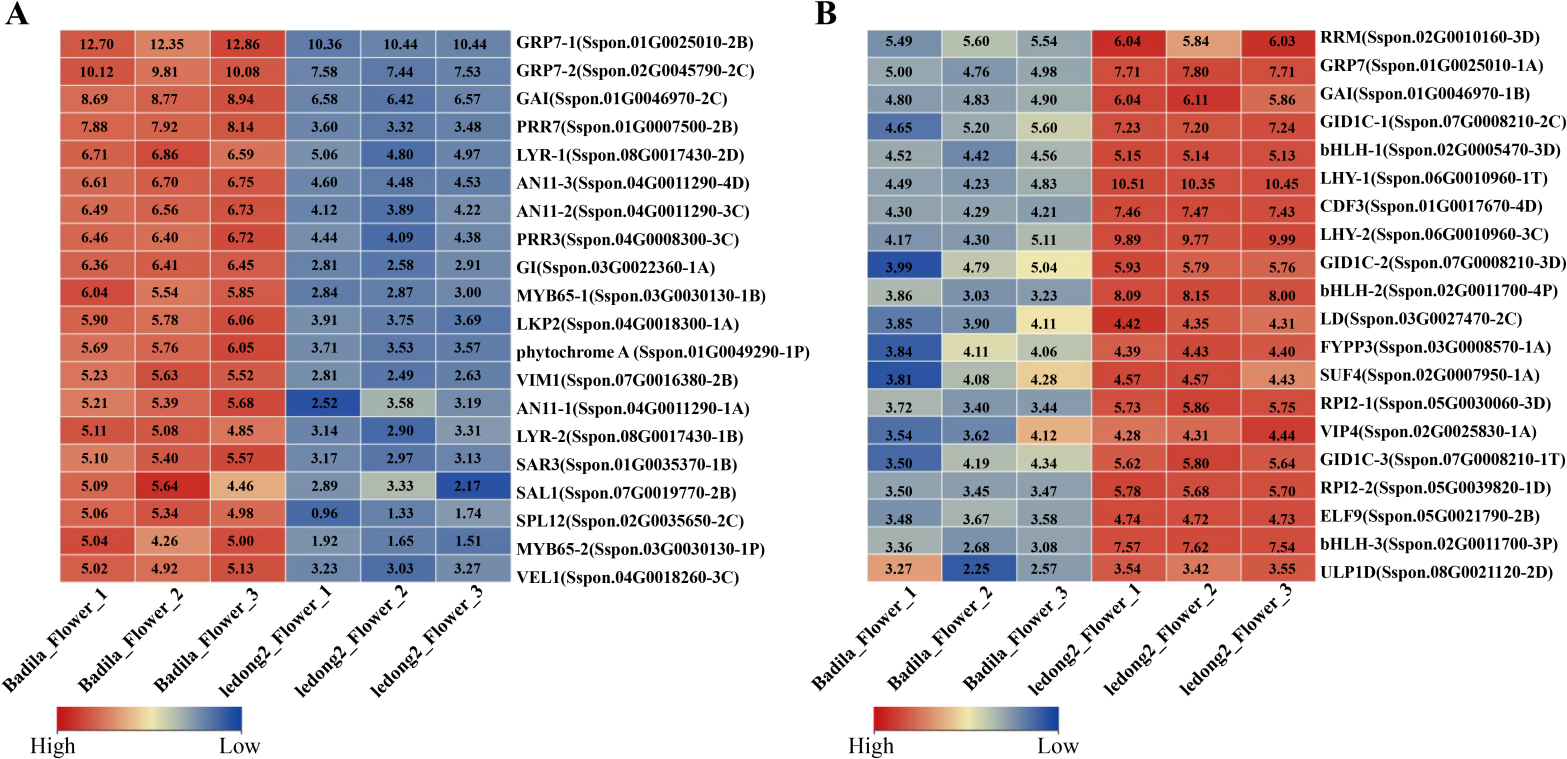
Different transcript expression levels of differentially expressed genes (DEGs) in flower between Badila and Ledong2. **(A, B)** Higher and lower expression levels (log_2_FC) of DEGs in Badila compared with Ledong2, respectively.

The clone Ledong2 is prone to flowering in Sayan, Hainan Province, China, with a pollen germination rate of approximately 95%. In contrast, Badila exhibits sporadic flowering and a significantly lower pollen germination rate (**Table 1**). To further explore the possible mechanisms associated with flowering differences between Badila and Ledong2, the expression levels of DEGs were identified. A total of 10,823 DEGs were identified in both clones (**Supplementary Figure S6** and **Supplementary Table S7**). In the photoperiodic flowering pathway, DEGs related to circadian rhythm exhibited notable variations between the two clones. The temperature-sensitive circadian gene *Pseudo-response regulator 7* (*PRR7*, *Sspon.01G0007500-2B*), which serves as a transcriptional repressor of *CIRCADIAN CLOCK ASSOCIATED1* (*CCA1*) and *LATE ELONGATED HYPOCOTYL* (*LHY*), was expressed at a lower level in Ledong2 compared with Badila (**Figure 5A**). Additionally, two *LHY* genes (*Sspon.06G0010960-1T* and *Sspon.06G0010960-3C*) were expressed at higher levels in Ledong2 than in Badila (**Figure 5B**). The *glycine-rich RNA-binding* gene (ScGRP7, *Sspon.01G0025010-1A*), which regulates circadian oscillations in plants and enhances cold tolerance, was expressed at a higher level in Ledong2 (**Figure 5B**). Three bHLH family genes (*Sspon.02G0011700-3P*, *Sspon.02G0011700-4P*, and *Sspon.02G0005470-3D*) involved in floral development were also expressed at higher levels in Ledong2 (**Figure 5B**). The MYB transcription factor (TF) genes (*MYB65*, *Sspon.03G0030130-1B*, and *Sspon.03G0030130-1P*) involved in pollen development were expressed at lower levels in Ledong2 (**Figure 5A**). Furthermore, genes encoding the *Gibberellin receptor* (*ScGID1*, *Sspon.07G0008210-1T*, *Sspon.07G0008210-2C*, and *Sspon.07G0008210-3D*), which are involved in floral organ morphogenesis, were expressed at higher levels in Ledong2 (**Figure 5B**).

**Table 1:** Flowering characteristics of two clones of Ledong2 (*Saccharum* sp*ontaneum*) and Badila (*Saccharum officinarum*) in Sayan, China.

Sugarcane clone	Year	Flowering date	Pollen amount	Pollen germination rate (%)
Ledong2	2018	Later September	Middle	98
	2019	Later September	Much	95
	2020	Early October	Middle	92
Badila	2018	Later January/second year	Less	10
	2019	Not flowering	No pollen	0
	2020	Not flowering	No pollen	0

### 3.7 Comparison of expression levels of key genes in different tissues between RNA-seq and RT-qPCR

**Figure 6:**
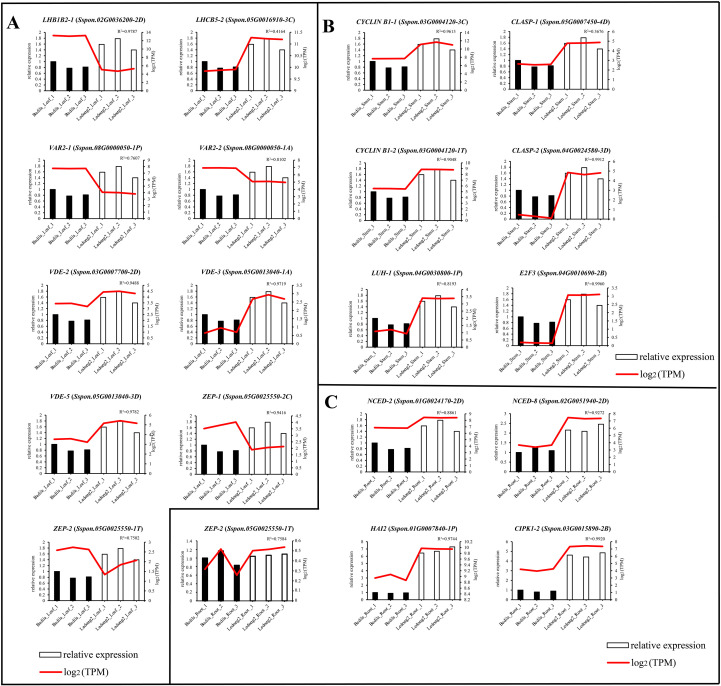
Comparisons of 19 representative genes’ expression levels in different tissues in Badila and Ledong2 determined using RNA sequencing (RNA-seq) and RT-qPCR datasets. **(A)** Leaf. **(B)** Stem. **(C)** Root. The gene expression level was determined using RNA-seq and RT-qPCR datasets with log_2_FC and 2^−ΔΔCt^ methods, respectively.

To further verify the reliability of the RNA-seq data obtained in our study, we selected 19 representative genes that are involved in leaf photosynthesis (nine genes in **Figure 6A**), stem development (six genes in **Figure 6B**), and root stress response (four genes in **Figure 6C**) and conducted RT-qPCR assays. Based on RNA-seq data, *LHB1B2-1* (Sspon.02G0036200-2D), *VAR2-1* (Sspon.08G0000050-1P), *VAR2-2* (Sspon.08G0000050-1A), *ZEP-1* (Sspon.05G0025550-2C), and *ZEP-2* (Sspon.05G0025550-1T) were expressed at lower levels in Ledong2, while other genes were expressed at higher levels in Ledong2. The results of RT-qPCR experiments corroborated the trends observed in the transcriptomic data, indicating that the expression patterns of these genes were generally consistent between both methods. Notably, all the 19 genes were expressed at lower levels in Badila than in Ledong2 (**Figures 6A–C**).

## 4 Discussion

The nobilization breeding process in sugarcane was carried out through pioneer crosses between *S. officinarum* and *S.* sp*ontaneum* having complementary traits on sugar, yield, and stress tolerance, generating the modern commercial cultivars with high levels of polyploidy and aneuploidy (Yu et al., 2018). Sugarcane genetic improvement is commonly generated from a great number of crosses by conventional hybrid breeding and consequently gives rise to the improved yield (biomass production and/or sugar content) and tolerance to environmental stress (McCormick et al., 2009; Calderan-Rodrigues et al., 2021). However, sugar content and cane yield seem to have reached a plateau due to modern sugarcane hybrid parents having a narrow germplasm genetic basis and the compromise with biotic/abiotic stress tolerance, in addition to the complex polyploid nature of this crop hampering the use of molecular breeding tools (Calderan-Rodrigues et al., 2021). Accurate evaluation of sugarcane germplasm materials is helping to mine more valuable gene sources for application in conventional and molecular sugarcane breeding. In this study, we identified a substantial number of DEGs that are involved in stress tolerance in root systems, cell division and growth in stems, photosynthesis in leaves, and flowering processes. These genes played important roles in growth and development, and stress response in sugarcane.

The ABA plays a significant role in modulating plant stress responses and regulating downstream signaling pathways (Chen et al., 2020; Wei et al., 2025). Some key genes, such as *ZEP*, *NECD*, *ABA2*, and *AAO3*, involved in ABA biosynthesis and CYP709B related to ABA catabolism, showed higher expression levels in Ledong2 than in Badila. ABA concentration in Ledong2 was significantly higher than that in Badila. Similarly, some genes, such as *ABF2*, involved in the ABA signaling transduction pathway, were upregulated in Ledong2 compared with Badila. These results indicated that ABA likely plays an important role in Ledong2 by affecting the expression of genes related to ABA biosynthesis and catabolism to control endogenous ABA levels in Ledong2. Additionally, many TFs, including WRKY33-1/2, NAC100, ONAC071, and MYC2, together with some protein kinases (MAPKKKK16 and CIPK1-2), had also higher levels in Ledong2. A study by Krishnamurthy et al. revealed that AtWRKY33-mediated regulation of AtCYP94B1 controlled apoplastic barrier formation in *Arabidopsis* roots to confer salt tolerance (Krishnamurthy et al., 2020). The SmMYC2, a key TF in the JA signaling pathway, functioned as a positive regulator to enhance salt resistance in *Arabidopsis thaliana* and *Salvia miltiorrhiza* hairy roots (Deng et al., 2023). These findings suggest that ABA and some important genes associated with stress responses play key roles in the *S.* sp*ontaneum* clone Ledong2 compared with the *S. officinarum* clone Badila. It is well known that the *S.* sp*ontaneum* clone is the main donor of resistance and adaptive genes in the nobilization breeding process (Jiang et al., 2023).

Based on the theory of source–sink relationships, sugarcane stem is a main source for biomass production and sucrose accumulation export by photosynthesizing leaves (source), and sugar allocation and import by sink (stems) tissues (Chen et al., 2024). Sugars are transported from source leaves to sink stems, triggering cell growth, elongation, and maintenance, which are regulated by a wide range of genes (Mehdi et al., 2024). *S. officinarum* clones have high sucrose content, low fiber content, and long and thick stems, whereas *S.* sp*ontaneum* clones have low sucrose content, high fiber content, and slender stems (Jiang et al., 2023; Thirugnanasambandam et al., 2023). In this study, numerous DEGs related to the regulation of cell growth and cell division were enriched in stems of both clones, suggesting that these DEGs play important roles in sugarcane stem growth and development, for example, the *ERECTA* family genes, *ERECTA* (*ER*), *ERECTA-LIKE1* (*ERL1*), and *ERECTA-LIKE2* (*ERL2*) encoding leucine-rich repeat receptor-like kinases, the cytoplasmic linker-associated proteins (CLASPs) known as microtubule-associated proteins, the CCCH-type zinc finger protein, and the transcription factor E2F3. A review by Jiang et al. summarized the essential roles of the *ERECTA* family genes in plant organ morphogenesis, including shoot apical meristem, stem, and reproductive organ development (Jiang et al., 2022). The CLASPs are involved in the regulation of the dynamics of microtubules that play an important role in plant growth and development (Takahiro, 2014). Overexpressing *GhCLASP2* can increase fiber strength in transgenic cotton (Takahiro, 2014). CLASP was translationally regulated in light-dependent root apical meristem growth (Halat et al., 2020) and balanced two competing cell division mechanisms to specify the distribution of cells and intercellular spaces in *Arabidopsis* spongy mesophyll (Zhang and Ambrose, 2022). CCCH-type zinc finger proteins participated in the anther development in rice (Wang et al., 2020) and pollen development in *Brassica campestris* ssp. *chinensis* (Xu et al., 2020).

Sugarcane is a typical C_4_ plant that possesses high biomass production, which depends on the high efficiency in assimilating carbon dioxide into carbohydrate by conversion of solar energy and avoiding photorespiration during daytime photosynthesis (Jiang et al., 2023). Our data revealed that, compared with Ledong2 (*S.* sp*ontaneum*), Badila (*S. officinarum*) had a higher light capture ability and less light energy dissipation in PS II yield, and a higher efficiency of carbon fixation through PS II under a diurnal rhythm. Accordingly, most DEGs related to light-harvesting chlorophyll *a*/*b*-binding (LHC) proteins are integral to the formation of thylakoid membrane structure and photosynthesis (Ye et al., 2024); VARIEGATED2 (VAR2) is encoded by filamentation temperature-sensitive H (FtsH) (an ATP-dependent metalloprotease in thylakoid membranes, which degrades several chloroplastic proteins) (Miura et al., 2007; Wang et al., 2022b). In *Arabidopsis*, VAR2/AtFtsH2 is required for the degradation of PS II subunits in cotyledons under photoinhibition (Qi et al., 2020; Wang et al., 2022b). Two key enzymes, such as ZEP and VDE in the xanthophyll cycle, protect photosynthesis apparatus from the damage of excessive light (Short et al., 2023); PETC provides energy and redox equivalents for efficient carbon fixation in the Calvin–Benson–Bassham cycle (Matuszyńska et al., 2019); and the CCB pathway [cofactor assembly, cytochrome b6f complex (Cyt b_6_f), and photosynthetic electron transport subunit B (PetB)] deliver heme to the cytochrome b6 apoprotein (Lezhneva et al., 2008; Sandoval-Ibáñez et al., 2022). Notably, the cytochrome b6f complex controls cyclic electron flow and photoprotection under limiting light and controls carbon metabolism under saturating light (Johnson and Berry, 2021).

Similarly, a comparable transcriptome analysis by Jiang et al. (2023) showed that most DEGs were enriched in photosynthesis and differential regulation of photosynthesis-related genes between *S. officinarum* and *S.* sp*ontaneum*. Their work also demonstrated that *S. officinarum* has a more active carbohydrate metabolism in the source tissue and less sensitivity to the diurnal rhythm than *S.* sp*ontaneum* (Jiang et al., 2023). In our study, Badila had higher biomass production than Ledong2 at the single plant scale. The reason is likely that Badila exhibits high efficiency in intercepting solar energy and assimilating carbon into carbohydrates, as supported by the phenotypic data of higher Y(II), IK, and ETR but lower NPQ in Badila than in Ledong2. In addition, the transcript expression levels of these DEGs are related to photosynthetic efficiency, electron transport ratio, and xanthophyll cycle-dependent photoprotection. Jiang et al. (2023) proposed that the divergence of photosynthesis between the two *Saccharum* species is possibly caused by variation in the photorespiratory pathway. The overexpression of *VDE*, *photosystem II subunit S* (*PsbS*), and *ZEP* from *Arabidopsis* resulted in faster NPQ relaxation and higher photosynthetic efficiency in soybean under fluctuating light, which implies that accelerating recovery from photoprotection contributes to soybean photosynthesis and crop yield (Souza et al., 2022).

Flowering is a crucial factor for hybrid breeding during sugarcane improvement, but it is an adverse phenomenon for commercial cultivars, depleting the sucrose reserves from the stalks during the transition from the vegetative to reproductive stage. Different species of the *Saccharum* genus have erratic flowering nature due to their geographical origins spreading across geographical latitudes (Pavani et al., 2023). Numerous DEGs associated with photoperiodic flowering pathways were found in two *Saccharum* species in this study. For instance, some genes, such as *SQUAMOSA promoter-binding protein-like transcription factor 12* (*SPL12*), *MYB65*, *GI* (*GIGANTEA*), *PRR7*, and *VIM1*, were expressed at higher levels in Badila (male sterile). Conversely, some genes, such as *bHLH*, *LHY*, and *Cycling dof factor 3* (*CDF3*), were expressed at higher levels in Ledong2 (robust and viable pollen). In the circadian rhythms and photoperiod pathway of *Arabidopsis*, *CCA1*/*LHY*, as the morning-phased genes, are directly activated by light through the interaction between the PHYTOCHROME INTERACTING FACTOR3 (PIF3) and light-activated PHYs (especially PHYB). However, the PRR5/7/9 proteins act as negative regulators by suppressing CCA1/LHY, while, in the case of the evening loop, CCA1/LHY directly suppresses GI, which is a known circadian clock component primarily participating in the promotion of flowering (Pavani et al., 2023). It was observed that the LHY peak was postponed in the plants in the west of the field or beyond the wooden wall, even though sugarcane was planted in the same field, which evidenced that the change of field microenvironments may impact important agronomical traits such as flowering time, stalk weight, and number (Dantas et al., 2021). Our data suggested that the reason for hard flowering in Badila is likely that the LHY was inhibited by the PRR7 protein, thereby impairing the downregulation of LHY on GI protein. Other proteins, such as SPL12, CDF3, and bHLH TF, may participate in the divergence of flowering in two *Saccharum* species. However, more robust experiments in sugarcane need to be identified to gain insight into the complete perspective driving flowering mechanisms.

## 5 Conclusion

This study employed transcriptome sequencing to investigate the DEGs in four distinct tissues (root, stem, leaf, and flower) of two *Saccharum* clones (Badila and Ledong2). Comparative transcriptome analysis revealed a diverse array of DEGs across various tissues in both clones. In the root tissue, hundreds of DEGs involved in ABA biosynthesis and signal transduction were identified, suggesting that the classical phytohormone ABA may play a role in sugarcane root morphogenesis and/or stress responses. Most of the DEGs involved in the regulation of cell growth and division (such as ERECTA-like2 genes) were found in the stem, which possibly explains the divergence of stem morphology between the two *Saccharum* species. DEGs associated with enhanced photosynthetic and carbon fixation efficiency were prevalent in leaf tissues, potentially explaining the differences in biomass production between Badila and Ledong2 at the individual plant level. Additionally, numerous key DEGs involved in the circadian rhythms and photoperiod pathway were significantly enriched in the flower tissues, offering valuable insights into the divergence of flowering characteristics in both clones. The identification of these key genes in diverse tissues paves the way for elucidating the morphogenetic and stress-response traits that differentiate the two *Saccharum* species. However, it is imperative to conduct more comprehensive studies that integrate the findings from transcriptome analysis with functional genomics to fully understand the underlying mechanisms.

## Data Availability

The datasets presented in this study can be found in online repositories. The names of the repository/repositories and accession number(s) can be found in the article/**Supplementary Material**.

## Supplementary material

Supplementary Figure 1 Gene expression (log_2_TPM) in four tissues including root, stem, leaf, and flower between Badila and Ledong2. TPM: transcripts per million.

Supplementary Figure 2 Correlation coefficient of gene expression (log_2_TPM) among 24 samples from four tissues root, stem, leaf, and flower between Badila and Ledong2.

Supplementary Figure 3 Gene ontology (GO) enrichment of differentially expressed genes (DEGs) in root tissues from Badila and Ledong2. **(A)** Upregulated DEGs; **(B)** Downregulated DEGs. BP: Biological process; CC: cellular component; MF: Molecular function.

Supplementary Figure 4 Gene ontology (GO) enrichment of differentially expressed genes (DEGs) in stem tissues from Badila and Ledong2. **(A)** Upregulated DEGs; **(B)** Downregulated DEGs. BP: Biological process; CC: cellular component; MF: Molecular function.

Supplementary Figure 5 Gene ontology (GO) enrichment of differentially expressed genes (DEGs) in leaf tissues from Badila and Ledong2. **(A)** Upregulated DEGs; **(B)** Downregulated DEGs. BP: Biological process; CC: cellular component; MF: Molecular function.

Supplementary Figure 6 Gene ontology (GO) enrichment of differentially expressed genes (DEGs) in flower tissues from Badila and Ledong2. **(A)** Upregulated DEGs; **(B)** Downregulated DEGs. BP: Biological process; CC: cellular component; MF: Molecular function.

Supplementary Figure 7 Top ten KEGG pathways in four tissues root **(A)**, stem **(B)**, leaf **(C)**, and flower **(D)** from Badila and Ledong2.

Supplementary Table 1 Nineteen primer pairs used in this study for reverse transcription-quantitative polymerase chain reaction (RT-qPCR) assay.

Supplementary Table 2 Summary of sequencing, assembly, and transcriptome analyses of 24 samples from two clones Badila and Ledong2.

Supplementary Table 3 Top ten KEGG pathways in four tissues root, stem, leaf, and flower from Badila and Ledong2.

Supplementary Table 4 Gene ontology (GO) enrichment of differentially expressed genes (DEGs) in root tissues from Badila and Ledong2.

Supplementary Table 5 Gene ontology (GO) enrichment of differentially expressed genes (DEGs) in stem tissues from Badila and Ledong2.

Supplementary Table 6 Gene ontology (GO) enrichment of differentially expressed genes (DEGs) in leaf tissues from Badila and Ledong2.

Supplementary Table 7 Gene ontology (GO) enrichment of differentially expressed genes (DEGs) in flowering tissues from Badila and Ledong2.

## References

[B1] Ali A. Khan M. Sharif R. Mujtaba M. Gao S.-J. (2019). Sugarcane omics: An update on the current status of research and crop improvement. Plants 8, 344. doi: 10.3390/plants8090344, PMID: 31547331 PMC6784093

[B2] Anders S. Pyl P. T. Huber W. (2015). HTSeq—a python framework to work with high-throughput sequencing data. Bioinformatics 31, 166–169. doi: 10.1093/bioinformatics/btu638, PMID: 25260700 PMC4287950

[B3] Aono A. H. Pimenta R. J. G. Garcia A. L. B. Correr F. H. Hosaka G. K. Carrasco M. M.. (2021). The wild sugarcane and sorghum kinomes: Insights into expansion, diversification, and expression patterns. Front. Plant Sci. 12. doi: 10.3389/fpls.2021.668623, PMID: 34305969 PMC8294386

[B4] Calderan-Rodrigues M. J. de Barros Dantas L. L. Cheavegatti Gianotto A. Caldana C. (2021). Applying molecular phenotyping tools to explore sugarcane carbon potential. Front. Plant Sci. 12. doi: 10.3389/fpls.2021.637166, PMID: 33679852 PMC7935522

[B5] Chen X.-W. Deng H.-H. Chen Y.-S. (2010). Utilization of Badila in the breeding of YC-series parents and new varieties of sugarcane. Sugarcane Canesugar 6), 1–5, 43. doi: 10.3969/j.issn.1005-9695.2010.06.001

[B6] Chen L. Ghannoum O. Furbank R. T. (2024). Sugar sensing in c4 source leaves: A gap that needs to be filled. J. Exp. Bot. 75, 3818–3834. doi: 10.1093/jxb/erae166, PMID: 38642398 PMC11233418

[B7] Chen K. Li G.-J. Bressan R. A. Song C.-P. Zhu J.-K. Zhao Y. (2020). Abscisic acid dynamics, signaling, and functions in plants. J. Integr. Plant Biol. 62, 25–54. doi: 10.1111/jipb.12899, PMID: 31850654

[B8] Chen M. Liu P. An R. He X. Zhao P. Huang D.. (2025). Sugarcane pan-transcriptome identifying a master gene ScHCT regulating lignin and sugar traits. J. Agric. Food Chem. 73, 1739–1755. doi: 10.1021/acs.jafc.4c10101, PMID: 39761552

[B9] D’Hont A. (2005). Unraveling the genome structure of polyploids using FISH and GISH; examples of sugarcane and banana. Cytogenet. Genome Res. 109, 27–33. doi: 10.1159/000082378, PMID: 15753555

[B10] Dantas L. L. B. Dourado M. M. Lima N. O. Cavaçana N. Nishiyama M. Y. Souza G. M.. (2021). Field microenvironments regulate crop diel transcript and metabolite rhythms. New Phytol. 232, 1738–1749. doi: 10.1111/nph.17650, PMID: 34312886

[B11] Deng H. Li Q. Cao R. Ren Y. Wang G. Guo H.. (2023). Overexpression of SmMYC2 enhances salt resistance in arabidopsis thaliana and salvia miltiorrhiza hairy roots. J. Plant Physiol. 280, 153862. doi: 10.1016/j.jplph.2022.153862, PMID: 36399834

[B12] Deng H.-H. Li Q.-W. Chen Z.-Y. (2004). Breeding and utilization of new sugarcane parents. Sugarcane 11, 7–12. doi: 10.3969/j.issn.1673-0925.2004.03.002

[B13] Garsmeur O. Droc G. Antonise R. Grimwood J. Potier B. Aitken K.. (2018). A mosaic monoploid reference sequence for the highly complex genome of sugarcane. Nat. Commun. 9, 2638. doi: 10.1038/s41467-018-05051-5, PMID: 29980662 PMC6035169

[B14] Halat L. Gyte K. Wasteneys G. (2020). The microtubule-associated protein CLASP is translationally regulated in light-dependent root apical meristem growth. Plant Physiol. 184, 2154–2167. doi: 10.1104/pp.20.00474, PMID: 33023938 PMC7723079

[B15] Jiang H. Chen Y. Liu Y. Shang J. Sun X. Du J. (2022). Multifaceted roles of the ERECTA family in plant organ morphogenesis. J. Exp. Bot. 73, 7208–7218. doi: 10.1093/jxb/erac353, PMID: 36056777

[B16] Jiang Q. Hua X. Shi H. Liu J. Yuan Y. Li Z.. (2023). Transcriptome dynamics provides insights into divergences of the photosynthesis pathway between saccharum officinarum and saccharum spontaneum. Plant J. 113, 1278–1294. doi: 10.1111/tpj.16110, PMID: 36648196

[B17] Johnson J. E. Berry J. A. (2021). The role of cytochrome b6f in the control of steady-state photosynthesis: A conceptual and quantitative model. Photosynth. Res. 148, 101–136. doi: 10.1007/s11120-021-00840-4, PMID: 33999328 PMC8292351

[B18] Khan Q. Chen J.-Y. Zeng X.-P. Qin Y. Guo D.-J. Mahmood A.. (2021). Transcriptomic exploration of a high sucrose mutant in comparison with the low sucrose mother genotype in sugarcane during sugar accumulating stage. GCB Bioenergy 13, 1448–1465. doi: 10.1111/gcbb.12868

[B19] Kim D. Langmead B. Salzberg S. L. (2015). HISAT: A fast spliced aligner with low memory requirements. Nat. Methods 12, 357–360. doi: 10.1038/nmeth.3317, PMID: 25751142 PMC4655817

[B20] Kolde R. Kolde M. R. (2015). Package ‘pheatmap’. R packag. 1, 18. doi: 10.32614/CRAN.package.pheatmap

[B21] Krishnamurthy P. Vishal B. Ho W. J. Lok F. C. J. Lee F. S. M. Kumar P. P. (2020). Regulation of a cytochrome p450 gene CYP94B1 by WRKY33 transcription factor controls apoplastic barrier formation in roots to confer salt tolerance. Plant Physiol. 184, 2199–2215. doi: 10.1104/pp.20.01054, PMID: 32928900 PMC7723105

[B22] Lezhneva L. Kuras R. Ephritikhine G. de Vitry C. (2008). A novel pathway of cytochrome c biogenesis is involved in the assembly of the cytochrome b6f complex in *Arabidopsis* chloroplasts. J. Biol. Chem. 283, 24608–24616. doi: 10.1074/jbc.M803869200, PMID: 18593701 PMC3259826

[B23] Li P. Yang R. Liu J. Huang C. Huang G. Deng Z.. (2025). Coexpression regulation of new and ancient genes in the dynamic transcriptome landscape of stem and rhizome development in “bainianzhe”—an ancient Chinese sugarcane variety ratooned for nearly 300 years. Plant Cell Environ. 48, 1621–1642. doi: 10.1111/pce.15232, PMID: 39462914

[B24] Loggini B. Scartazza A. Brugnoli E. Navari-Izzo F. (1999). Antioxidative defense system, pigment composition, and photosynthetic efficiency in two wheat cultivars subjected to drought. Plant Physiol. 119, 1091–1100. doi: 10.1104/pp.119.3.1091, PMID: 10069848 PMC32091

[B25] Manechini J. R. V. Santos P. H. da S. Romanel E. dos S. M. Scarpari M. S.. (2021). Transcriptomic analysis of changes in gene expression during flowering induction in sugarcane under controlled photoperiodic conditions. Front. Plant Sci. 12. doi: 10.3389/fpls.2021.635784, PMID: 34211482 PMC8239368

[B26] Matuszyńska A. Saadat N. P. Ebenhöh O. (2019). Balancing energy supply during photosynthesis – a theoretical perspective. Physiol. Plant 166, 392–402. doi: 10.1111/ppl.12962, PMID: 30864189 PMC6849747

[B27] Maxwell K. Johnson G. N. (2000). Chlorophyll fluorescence—a practical guide. J. Exp. Bot. 51, 659–668. doi: 10.1093/jexbot/51.345.659 10938857

[B28] McCormick A. J. Watt D. A. Cramer M. D. (2009). Supply and demand: Sink regulation of sugar accumulation in sugarcane. J. Exp. Bot. 60, 357–364. doi: 10.1093/jxb/ern310, PMID: 19050062

[B29] Mehdi F. Galani S. Wickramasinghe K. P. Zhao P. Lu X. Lin X.. (2024). Current perspectives on the regulatory mechanisms of sucrose accumulation in sugarcane. Heliyon 10, e27277. doi: 10.1016/j.heliyon.2024.e27277, PMID: 38463882 PMC10923725

[B30] Miura E. Kato Y. Matsushima R. Albrecht V. Laalami S. Sakamoto W. (2007). The balance between protein synthesis and degradation in chloroplasts determines leaf variegation in arabidopsis yellow variegated mutants. Plant Cell 19, 1313–1328. doi: 10.1105/tpc.106.049270, PMID: 17416734 PMC1913758

[B31] Pavani G. Malhotra P. K. Verma S. K. (2023). Flowering in sugarcane-insights from the grasses. 3 Biotech. 13, 154. doi: 10.1007/s13205-023-03573-4, PMID: 37138783 PMC10149435

[B32] Qi Y. Wang X. Lei P. Li H. Yan L. Zhao J.. (2020). The chloroplast metalloproteases VAR2 and EGY1 act synergistically to regulate chloroplast development in arabidopsis. J. Biol. Chem. 295, 1036–1046. doi: 10.1016/S0021-9258(17)49913-3, PMID: 31836664 PMC6983840

[B33] Robinson M. D. McCarthy D. J. Smyth G. K. (2010). EdgeR: A bioconductor package for differential expression analysis of digital gene expression data. Bioinf. 26, 139–140. doi: 10.1093/bioinformatics/btp616, PMID: 19910308 PMC2796818

[B34] Sandoval-Ibáñez O. Rolo D. Ghandour R. Hertle A. P. Armarego-Marriott T. Sampathkumar A.. (2022). De-etiolation-induced protein 1 (DEIP1) mediates assembly of the cytochrome b6f complex in arabidopsis. Nat. Commun. 13, 4045. doi: 10.1038/s41467-022-31758-7, PMID: 35831297 PMC9279372

[B35] Short A. Fay T. P. Crisanto T. Mangal R. Niyogi K. K. Limmer D. T.. (2023). Kinetics of the xanthophyll cycle and its role in photoprotective memory and response. Nat. Commun. 14, 6621. doi: 10.1038/s41467-023-42281-8, PMID: 37857617 PMC10587229

[B36] Song J. Zhang X. Jones T. Wang M.-L. Ming R. (2024). Identification of male sterility-related genes in *Saccharum officinarum* and *Saccharum* sp*ontaneum* . Plant Reprod. 37, 489–506. doi: 10.1007/s00497-024-00503-z, PMID: 38844561

[B37] Souza A. P. D. Burgess S. J. Doran L. Hansen J. Manukyan L. Maryn N.. (2022). Soybean photosynthesis and crop yield are improved by accelerating recovery from photoprotection. Science 377, 851–854. doi: 10.1126/science.adc9831, PMID: 35981033

[B38] Takahiro H. (2014). Microtubule organization and microtubule-associated proteins in plant cells. Int. Rev. Cell Mol. Biol. 312, 1–52. doi: 10.1016/B978-0-12-800178-3.00001-4, PMID: 25262237

[B39] Thimm O. Bläsing O. Gibon Y. Nagel A. Meyer S. Krüger P.. (2004). Mapman: A user-driven tool to display genomics data sets onto diagrams of metabolic pathways and other biological processes. Plant J. 37, 914–939. doi: 10.1111/j.1365-313X.2004.02016.x, PMID: 14996223

[B40] Thirugnanasambandam P. P. Singode A. Thalambedu L. P. Athiappan S. Krishnasamy M. Purakkal S. V.. (2023). Long read transcriptome sequencing of a sugarcane hybrid and its progenitors, *Saccharum officinarum* and *S.* sp*ontaneum* . Front. Plant Sci. 14. doi: 10.3389/fpls.2023.1199748, PMID: 37662143 PMC10469502

[B41] Wang B. Fang R. Chen F. Han J. Liu Y.-G. Chen L.. (2020). A novel CCCH-type zinc finger protein SAW1 activates OsGA20ox3 to regulate gibberellin homeostasis and anther development in rice. J. Integr. Plant Biol. 62, 1594–1606. doi: 10.1111/jipb.12924, PMID: 32149461

[B42] Wang T. Fang J. Zhang J. (2022a). Advances in sugarcane genomics and genetics. Sugar Tech 24, 354–368. doi: 10.1007/s12355-021-01065-4

[B43] Wang X. Li Q. Zhang Y. Pan M. Wang R. Sun Y.. (2022b). VAR2/AtFtsH2 and EVR2/BCM1/CBD1 synergistically regulate the accumulation of PSII reaction centre d1 protein during de-etiolation in *Arabidopsis* . Plant Cell Environ. 45, 2395–2409. doi: 10.1111/pce.14368, PMID: 35610189

[B44] Wei Y.-S. Javed T. Liu T.-T. Ali A. Gao S.-J. (2025). Mechanisms of abscisic acid (ABA)-mediated plant defense responses: An updated review. Plant Stress 15, 100724. doi: 10.1016/j.stress.2024.100724

[B45] Wu K.-C. Huang C.-M. Verma K. K. Deng Z.-N. Huang H.-R. Pang T.. (2022). Transcriptomic responses of *Saccharum* spontaneum roots in response to polyethylene glycol – 6000 stimulated drought stress. Front. Plant Sci. 13. doi: 10.3389/fpls.2022.992755, PMID: 36352884 PMC9638123

[B46] Xu L. Liu T. Xiong X. Liu W. Yu Y. Cao J. (2020). Overexpression of two CCCH-type zinc-finger protein genes leads to pollen abortion in *Brassica campestris* ssp. *chinensis* . Genes 11, 1287. doi: 10.3390/genes11111287, PMID: 33138166 PMC7693475

[B47] Yang Y. Saand M. A. Huang L. Abdelaal W. B. Zhang J. Wu Y.. (2021). Applications of multi-omics technologies for crop improvement. Front. Plant Sci. 12. doi: 10.3389/fpls.2021.563953, PMID: 34539683 PMC8446515

[B48] Ye J.-J. Lin X.-Y. Yang Z.-X. Wang Y.-Q. Liang Y.-R. Wang K.-R.. (2024). The light-harvesting chlorophyll a/b-binding proteins of photosystem II family members are responsible for temperature sensitivity and leaf color phenotype in albino tea plant. J. Adv. Res. 66, 87–104. doi: 10.1016/j.jare.2023.12.017, PMID: 38151116 PMC11674787

[B49] Yu G. Wang L.-G. Han Y. He Q.-Y. (2012). ClusterProfiler: An R package for comparing biological themes among gene clusters. OMICS: J. Integr. Biol. 16, 284–287. doi: 10.1089/omi.2011.0118, PMID: 22455463 PMC3339379

[B50] Yu F. Wang P. Li X. Huang Y. Wang Q. Luo L.. (2018). Characterization of chromosome composition of sugarcane in nobilization by using genomic in *situ* hybridization. Mol. Cytogenet. 11, 35. doi: 10.1186/s13039-018-0387-z, PMID: 29977338 PMC5992832

[B51] Zhang L. Ambrose C. (2022). CLASP balances two competing cell division plane cues during leaf development. Nat. Plants 8, 682–693. doi: 10.1038/s41477-022-01163-5, PMID: 35668154

[B52] Zhang J. Zhang X. Tang H. Zhang Q. Hua X. Ma X.. (2018). Allele-defined genome of the autopolyploid sugarcane *Saccharum* sp*ontaneum* L. Nat. Genet. 50, 1565–1573. doi: 10.1038/s41588-018-0237-2, PMID: 30297971

