# Supplementary material for: Differential gene expression and phenotypic variation across tissues between Saccharum officinarum and Saccharum spontaneum
Source: Front Plant Sci. 2025 Oct 31;16:1696921. doi: 10.3389/fpls.2025.1696921 (PMC12617224; doi:10.3389/fpls.2025.1696921)
Supplement: Supplementary Figure 1 — Gene expression (log2TPM) in four tissues including root, stem, leaf, and flower between Badila and Ledong2. TPM: transcripts per million. [file DataSheet1.zip › Supplement information-0901/Supplementary Table S1 Primers-20240709.docx]

**Table S1**. Nineteen primer pairs used in this study for reverse transcription-quantitative polymerase chain reaction (RT-qPCR) assay.

| **Gene name** | **Gene ID** | **Primer name** | **Primer sequence (5'-3')** |
| --- | --- | --- | --- |
| NCED-2 | Sspon.01G0024170-2D | NCED-2-F2 | TCAAGAAGCCGTACCTCAAGTA |
|  |  | NCED-2-R2 | CATCTCCTGGAGCTTGAACAC |
| NCED-8 | Sspon.02G0051940-2D | NCED-8-F1 | TCAAGCTCCAGGAGATGGTG |
|  |  | NCED-8-R1 | TCCAGAGGTGGAAGCAGAAG |
| HAI2 | Sspon.01G0007840-1P | HAI2-F1 | GAGCTACTCGAGGATGGACG |
|  |  | HAI2-R1 | TCATCTGAGAGAGGGATCGC |
| CIPK1-2 | Sspon.03G0015890-2B | CIPK1-2-F5 | GTTCCATATGATGATGATGAAGACG |
|  |  | CIPK1-2-R5 | CTCGAATAAACCAGAAAGATCAAGG |
| LHB1B2-1 | Sspon.02G0036200-2D | LHB1B2-1-F1 | GTGAAGGAGCTCAAGAACGG |
|  |  | LHB1B2-1-R1 | GTTGCCGGGAACGAAGTTAG |
| LHCB5-2 | Sspon.05G0016910-3C | LHCB5-2-F1 | CAACAGCATCCCCATCAACC |
|  |  | LHCB5-2-R1 | GACGTAGGCCTGGATGAAGA |
| VAR2-1 | Sspon.08G0000050-1P | VAR2-1-F2 | GTGCTTCCCTTCTCAGTACTTAC |
|  |  | VAR2-1-R2 | AAGGAACCTCGAATAAGACATCC |
| VAR2-2 | Sspon.08G0000050-1A | VAR2-2-F1 | CCATGAGTCTTGCGGCAA |
|  |  | VAR2-2-R1 | TTGTGCTCCAAGGAAGCG |
| VDE-2 | Sspon.03G0007700-2D | VDE-2-F1 | AACCCTGGAAGGAGAACTGG |
|  |  | VDE-2-R1 | GAAATCCTCCGCAACGTCAA |
| VDE-3 | Sspon.05G0013040-1A | VDE-3-F1 | TTGGGGTGATGGCATGTG |
|  |  | VDE-3-R1 | GCATGCTACGTTTGCTGC |
| VDE-5 | Sspon.05G0013040-3D | VDE-5-F3 | CACCTACCTTCTTAACCGCC |
|  |  | VDE-5-R3 | GTGATCCGGGGAGTATCTGA |
| ZEP-1 | Sspon.05G0025550-2C | ZEP-1-F3 | CGTTATGAGAAAGAGAGAAGGCT |
|  |  | ZEP-1-R3 | CATTTGCCTTATCAGAAAGTCGG |
| ZEP-2 | Sspon.05G0025550-1T | ZEP-2-F2 | TTGTGCCACCTGATATCGATAC |
|  |  | ZEP-2-R2 | CACCATCCGTCAAATATCTCGA |
| CYCLIN B1-1 | Sspon.03G0004120-3C | CYCLIN B1-1-F3 | GTATATCATCGATCAGTACCTCTCG |
|  |  | CYCLIN B1-1-R3 | CTGTATGCACTGTCTGATATAAGGA |
| CYCLIN B1-2 | Sspon.03G0004120-1T | CYCLIN B1-2-F3 | GGTCTTCTTCTTTGCTGAATTGG |
|  |  | CYCLIN B1-2-R3 | CTTCGTGCATTCAATTAGCTCTG |
| LUH | Sspon.04G0030800-1P | LUH-F1 | GCAACTGGGAAGCGGATAAG |
|  |  | LUH-R1 | CAATTGCGACTGGATCAGCA |
| CLASP-1 | Sspon.05G0007450-4D | CLASP-1-F2 | TTGCCTACATTTCTGCCAGC |
|  |  | CLASP-1-R2 | CCAGGCAGAACACAACAGTC |
| CLASP-2 | Sspon.04G0024580-3D | CLASP-2-F2 | TCTCCAAAGGCAAAACTGGC |
|  |  | CLASP-2-R2 | TAGGACTGCGGCTGAATCAA |
| E2F3 | Sspon.04G0010690-2B | E2F3-F1 | GGGATGACAAGGCTTACTGC |
|  |  | E2F3-R1 | CATATGTGGCCCGTCAACTG |
| GAPDH |  | GAPDH-F  GAPDH-R | CACGGCCACTGGAAGCA  TCCTCAGGGTTCCTGATGCC |
