# Supplementary material for: Differential gene expression and phenotypic variation across tissues between Saccharum officinarum and Saccharum spontaneum
Source: Front Plant Sci. 2025 Oct 31;16:1696921. doi: 10.3389/fpls.2025.1696921 (PMC12617224; doi:10.3389/fpls.2025.1696921)
Supplement: Supplementary Figure 1 — Gene expression (log2TPM) in four tissues including root, stem, leaf, and flower between Badila and Ledong2. TPM: transcripts per million. [file DataSheet1.zip › Supplement information-0901/Supplementary Table S2 Summary of sequencing-20240709.docx]

**Table S2.** Summary of sequencing, assembly, and transcriptome analyses of 24 samples from two clones Badila and Ledong2.

| **Sample** | **After_filtering_total_reads** | **After_filtering_total_bases** | **After_filtering_q20_bases** | **After_filtering_q30_bases** | **After_filtering_q20_rate** | **After_filtering_q30_rate** | **After_filtering_read1_mean_length** | **After_filtering_read2_mean_length** | **After_filtering_gc_content** |
| --- | --- | --- | --- | --- | --- | --- | --- | --- | --- |
| **Badila_Root_1** | 98152402 | 14628203932 | 14380527077 | 13895195199 | 0.983069 | 0.949891 | 149 | 149 | 0.564846 |
| **Badila_Root_2** | 98661354 | 14679680317 | 14447489143 | 13998073973 | 0.984183 | 0.953568 | 148 | 148 | 0.568701 |
| **Badila_Root_3** | 98688198 | 14718003618 | 14485690630 | 14027004895 | 0.984216 | 0.953051 | 149 | 149 | 0.564038 |
| **Badila_Flower_1** | 100564908 | 15000839479 | 14607229856 | 13919192072 | 0.973761 | 0.927894 | 149 | 149 | 0.540238 |
| **Badila_Flower_2** | 90194726 | 13449865192 | 13084074646 | 12442527304 | 0.972803 | 0.925104 | 149 | 149 | 0.541775 |
| **Badila_Flower_3** | 107793470 | 16083042764 | 15665415222 | 14934647391 | 0.974033 | 0.928596 | 149 | 149 | 0.537528 |
| **Badila_Stem_1** | 119336738 | 17827819529 | 17426503451 | 16685972101 | 0.977489 | 0.935951 | 149 | 149 | 0.550707 |
| **Badila_Stem_2** | 129131442 | 19277625024 | 18901084275 | 18192221394 | 0.980467 | 0.943696 | 149 | 149 | 0.556518 |
| **Badila_Stem_3** | 110017050 | 16421948381 | 16046651516 | 15369087690 | 0.977147 | 0.935887 | 149 | 149 | 0.55791 |
| **Badila_Leaf_1** | 102534938 | 15294152014 | 15041872030 | 14540591324 | 0.983505 | 0.950729 | 149 | 149 | 0.561405 |
| **Badila_Leaf_2** | 103846338 | 15496535469 | 15267846582 | 14809086875 | 0.985243 | 0.955639 | 149 | 149 | 0.556383 |
| **Badila_Leaf_3** | 88249242 | 13188537226 | 12951561038 | 12484775507 | 0.982032 | 0.946638 | 149 | 149 | 0.561873 |
| **Ledong2_Root_1** | 106278746 | 15858082217 | 15565308593 | 14994452638 | 0.981538 | 0.94554 | 149 | 149 | 0.559593 |
| **Ledong2_Root_2** | 84409290 | 12591253861 | 12396383358 | 12004851371 | 0.984523 | 0.953428 | 149 | 149 | 0.553018 |
| **Ledong2_Root_3** | 71286248 | 10632109447 | 10464818722 | 10132662849 | 0.984266 | 0.953025 | 149 | 149 | 0.558872 |
| **Ledong2_Flower_1** | 87478310 | 13025928810 | 12816758117 | 12404008170 | 0.983942 | 0.952255 | 148 | 148 | 0.548943 |
| **Ledong2_Flower_2** | 100563056 | 14954785492 | 14711844382 | 14234629723 | 0.983755 | 0.951844 | 148 | 148 | 0.554038 |
| **Ledong2_Flower_3** | 90395628 | 13478723281 | 13266847790 | 12851649689 | 0.984281 | 0.953477 | 149 | 149 | 0.549825 |
| **Ledong2_Stem_1** | 91616860 | 13651712955 | 13314023083 | 12702156503 | 0.975264 | 0.930444 | 149 | 148 | 0.552148 |
| **Ledong2_Stem_2** | 86490242 | 12894082233 | 12678064346 | 12251488480 | 0.983247 | 0.950164 | 149 | 149 | 0.557135 |
| **Ledong2_Stem_3** | 100061650 | 14928299780 | 14677486044 | 14182723179 | 0.983199 | 0.950056 | 149 | 149 | 0.549118 |
| **Ledong2_Leaf_1** | 90106342 | 13415817740 | 13176738933 | 12713747940 | 0.982179 | 0.947669 | 148 | 148 | 0.560415 |
| **Ledong2_Leaf_2** | 109721536 | 16337150534 | 16084984565 | 15582518359 | 0.984565 | 0.953809 | 148 | 148 | 0.552834 |
| **Ledong2_Leaf_3** | 91365346 | 13608300200 | 13388315848 | 12955185238 | 0.983835 | 0.952006 | 148 | 148 | 0.562786 |
