# Supplementary material for: Differential gene expression and phenotypic variation across tissues between Saccharum officinarum and Saccharum spontaneum
Source: Front Plant Sci. 2025 Oct 31;16:1696921. doi: 10.3389/fpls.2025.1696921 (PMC12617224; doi:10.3389/fpls.2025.1696921)
Supplement: Supplementary Figure 1 — Gene expression (log2TPM) in four tissues including root, stem, leaf, and flower between Badila and Ledong2. TPM: transcripts per million. [file DataSheet1.zip › Supplement information-0901/Supplementary Table S3 Top 10 KEGG pathways for each tissue.docx]

**Table S3** Top ten KEGG pathways in four tissues root, stem, leaf, and flower from Badila and Ledong2.

| Badila vs. Ledong2 Root upDEGs KEGG enrichment | | | | | | | |
| --- | --- | --- | --- | --- | --- | --- | --- |
| ID | Description | GeneRatio | BgRatio | pvalue | p.adjust | qvalue | Count |
| ko00983 | Drug metabolism - other enzymes | 0.0204299 | 0.010576094 | 1.99E-13 | 7.28E-11 | 6.02E-11 | 115 |
| ko00980 | Metabolism of xenobiotics by cytochrome P450 | 0.0181204 | 0.009617535 | 2.40E-11 | 4.37E-09 | 3.62E-09 | 102 |
| ko05204 | Chemical carcinogenesis | 0.0175875 | 0.009329968 | 4.53E-11 | 4.85E-09 | 4.01E-09 | 99 |
| ko00982 | Drug metabolism - cytochrome P450 | 0.0166992 | 0.008722881 | 5.31E-11 | 4.85E-09 | 4.01E-09 | 94 |
| ko05225 | Hepatocellular carcinoma | 0.0179428 | 0.009745343 | 1.33E-10 | 9.69E-09 | 8.02E-09 | 101 |
| ko00480 | Glutathione metabolism | 0.0206076 | 0.012557114 | 1.35E-08 | 8.20E-07 | 6.78E-07 | 116 |
| ko01524 | Platinum drug resistance | 0.0170545 | 0.009905103 | 1.80E-08 | 9.39E-07 | 7.77E-07 | 96 |
| ko00902 | Monoterpenoid biosynthesis | 0.008172 | 0.004153753 | 1.70E-06 | 7.75E-05 | 6.41E-05 | 46 |
| ko04910 | Insulin signaling pathway | 0.0170545 | 0.011151229 | 6.77E-06 | 0.00027446 | 0.000227 | 96 |
| ko03030 | DNA replication | 0.0110144 | 0.006646004 | 2.08E-05 | 0.000758959 | 0.000628 | 62 |
|  |  |  |  |  |  |  |  |
| Badila vs. Ledong2 Root downDEGs KEGG enrichment | | | | | | | |
| ID | Description | GeneRatio | BgRatio | pvalue | p.adjust | qvalue | Count |
| ko00010 | Glycolysis / Gluconeogenesis | 0.0286126 | 0.01511327 | 1.24E-14 | 4.55E-12 | 3.84E-12 | 139 |
| ko00710 | Carbon fixation in photosynthetic organisms | 0.0168794 | 0.008083842 | 1.52E-11 | 2.78E-09 | 2.34E-09 | 82 |
| ko00350 | Tyrosine metabolism | 0.0109098 | 0.004377416 | 4.05E-11 | 4.94E-09 | 4.16E-09 | 53 |
| ko00950 | Isoquinoline alkaloid biosynthesis | 0.0063812 | 0.002108828 | 1.72E-09 | 1.58E-07 | 1.33E-07 | 31 |
| ko00250 | Alanine, aspartate and glutamate metabolism | 0.0125566 | 0.006166725 | 1.66E-08 | 1.22E-06 | 1.03E-06 | 61 |
| ko00270 | Cysteine and methionine metabolism | 0.0183203 | 0.010767805 | 1.73E-07 | 1.06E-05 | 8.91E-06 | 89 |
| ko00220 | Arginine biosynthesis | 0.0067929 | 0.003227146 | 1.38E-05 | 0.000718048 | 0.000605 | 33 |
| ko00430 | Taurine and hypotaurine metabolism | 0.0039111 | 0.001437837 | 1.65E-05 | 0.000718048 | 0.000605 | 19 |
| ko00564 | Glycerophospholipid metabolism | 0.0193495 | 0.012780778 | 1.77E-05 | 0.000718048 | 0.000605 | 94 |
| ko00561 | Glycerolipid metabolism | 0.0150268 | 0.009681439 | 6.24E-05 | 0.00228495 | 0.001925 | 73 |
|  |  |  |  |  |  |  |  |
| Badila vs. Ledong2 Stem upDEGs KEGG enrichment | | | | | | | |
| ID | Description | GeneRatio | BgRatio | pvalue | p.adjust | qvalue | Count |
| ko03030 | DNA replication | 0.0169622 | 0.006646004 | 4.90E-32 | 1.77E-29 | 1.32E-29 | 132 |
| ko05322 | Systemic lupus erythematosus | 0.0114366 | 0.004473272 | 3.78E-22 | 6.81E-20 | 5.09E-20 | 89 |
| ko04110 | Cell cycle | 0.0219738 | 0.011406844 | 2.79E-21 | 3.36E-19 | 2.51E-19 | 171 |
| ko00194 | Photosynthesis proteins | 0.0128502 | 0.005687446 | 4.54E-19 | 4.10E-17 | 3.06E-17 | 100 |
| ko04914 | Progesterone−mediated oocyte maturation | 0.0091236 | 0.003578618 | 7.23E-18 | 5.22E-16 | 3.89E-16 | 71 |
| ko03440 | Homologous recombination | 0.0147777 | 0.007253091 | 4.71E-17 | 2.84E-15 | 2.12E-15 | 115 |
| ko00195 | Photosynthesis | 0.0101516 | 0.00444132 | 9.18E-16 | 4.73E-14 | 3.53E-14 | 79 |
| ko03460 | Fanconi anemia pathway | 0.0122077 | 0.006006966 | 2.93E-14 | 1.32E-12 | 9.87E-13 | 95 |
| ko04111 | Cell cycle − yeast | 0.0182472 | 0.010384382 | 8.06E-14 | 3.23E-12 | 2.41E-12 | 142 |
| ko03430 | Mismatch repair | 0.0106656 | 0.005272071 | 1.64E-12 | 5.93E-11 | 4.43E-11 | 83 |
|  |  |  |  |  |  |  |  |
| Badila vs. Ledong2 Stem downDEGs KEGG enrichment | | | | | | | |
| ID | Description | GeneRatio | BgRatio | pvalue | p.adjust | qvalue | Count |
| ko00360 | Phenylalanine metabolism | 0.0116546 | 0.005847206 | 1.03E-09 | 3.65E-07 | 3.24E-07 | 71 |
| ko00561 | Glycerolipid metabolism | 0.0162508 | 0.009681439 | 3.22E-08 | 5.72E-06 | 5.09E-06 | 99 |
| ko00410 | beta−Alanine metabolism | 0.0086999 | 0.004281561 | 6.03E-08 | 7.14E-06 | 6.35E-06 | 53 |
| ko00030 | Pentose phosphate pathway | 0.010998 | 0.006294533 | 1.03E-06 | 9.13E-05 | 8.12E-05 | 67 |
| ko00625 | Chloroalkane and chloroalkene degradation | 0.003283 | 0.001182222 | 2.98E-06 | 0.000211294 | 0.000188 | 20 |
| ko00592 | alpha−Linolenic acid metabolism | 0.0088641 | 0.004920599 | 3.93E-06 | 0.000232315 | 0.000207 | 54 |
| ko00400 | Phenylalanine, tyrosine and tryptophan biosynthesis | 0.0086999 | 0.004824744 | 4.64E-06 | 0.000235538 | 0.00021 | 53 |
| ko00903 | Limonene and pinene degradation | 0.0022981 | 0.000734895 | 1.51E-05 | 0.000594486 | 0.000529 | 14 |
| ko00981 | Insect hormone biosynthesis | 0.0022981 | 0.000734895 | 1.51E-05 | 0.000594486 | 0.000529 | 14 |
| ko04975 | Fat digestion and absorption | 0.0026264 | 0.000958558 | 3.66E-05 | 0.001299647 | 0.001156 | 16 |
|  |  |  |  |  |  |  |  |
| Badila vs. Ledong2 Leaf upDEGs KEGG enrichment | | | | | | | |
| ID | Description | GeneRatio | BgRatio | pvalue | p.adjust | qvalue | Count |
| ko04371 | Apelin signaling pathway | 0.0151515 | 0.007860178 | 9.93E-10 | 3.58E-07 | 2.79E-07 | 81 |
| ko04020 | Calcium signaling pathway | 0.0095398 | 0.004249609 | 4.19E-09 | 7.53E-07 | 5.86E-07 | 51 |
| ko04530 | Tight junction | 0.0160868 | 0.008818737 | 6.26E-09 | 7.53E-07 | 5.86E-07 | 86 |
| ko05410 | Hypertrophic cardiomyopathy (HCM) | 0.0082305 | 0.00361057 | 2.76E-08 | 2.49E-06 | 1.94E-06 | 44 |
| ko04921 | Oxytocin signaling pathway | 0.0136551 | 0.007412851 | 5.41E-08 | 3.91E-06 | 3.04E-06 | 73 |
| ko00195 | Photosynthesis | 0.0093528 | 0.00444132 | 6.68E-08 | 4.02E-06 | 3.13E-06 | 50 |
| ko00194 | Photosynthesis proteins | 0.0110363 | 0.005687446 | 1.34E-07 | 6.93E-06 | 5.40E-06 | 59 |
| ko00240 | Pyrimidine metabolism | 0.0218855 | 0.014058855 | 3.14E-07 | 1.42E-05 | 1.10E-05 | 117 |
| ko04068 | FoxO signaling pathway | 0.0119716 | 0.006709908 | 1.20E-06 | 4.59E-05 | 3.57E-05 | 64 |
| ko04710 | Circadian rhythm | 0.0102881 | 0.005495734 | 1.27E-06 | 4.59E-05 | 3.57E-05 | 55 |
|  |  |  |  |  |  |  |  |
| Badila vs. Ledong2 Leaf downDEGs KEGG enrichment | | | | | | | |
| ID | Description | GeneRatio | BgRatio | pvalue | p.adjust | qvalue | Count |
| ko00360 | Phenylalanine metabolism | 0.0111133 | 0.005847206 | 7.13E-07 | 0.00025543 | 0.000237 | 56 |
| ko00010 | Glycolysis / Gluconeogenesis | 0.022822 | 0.01511327 | 2.30E-06 | 0.000412508 | 0.000382 | 115 |
| ko00030 | Pentose phosphate pathway | 0.0107164 | 0.006294533 | 3.83E-05 | 0.004568541 | 0.004231 | 54 |
| ko00405 | Phenazine biosynthesis | 0.0015876 | 0.000383423 | 0.0001203 | 0.008614074 | 0.007978 | 8 |
| ko02025 | Biofilm formation − Pseudomonas aeruginosa | 0.0015876 | 0.000383423 | 0.0001203 | 0.008614074 | 0.007978 | 8 |
| ko00400 | Phenylalanine, tyrosine and tryptophan biosynthesis | 0.008335 | 0.004824744 | 0.0001855 | 0.011067496 | 0.010251 | 42 |
| ko04915 | Estrogen signaling pathway | 0.0073427 | 0.004121801 | 0.0002237 | 0.011443189 | 0.010599 | 37 |
| ko00520 | Amino sugar and nucleotide sugar metabolism | 0.0202421 | 0.014602039 | 0.0002926 | 0.013093265 | 0.012127 | 102 |
| ko00051 | Fructose and mannose metabolism | 0.0111133 | 0.007125284 | 0.0003492 | 0.013890156 | 0.012865 | 56 |
| ko05134 | Legionellosis | 0.0089303 | 0.005463782 | 0.0004337 | 0.015526014 | 0.01438 | 45 |
|  |  |  |  |  |  |  |  |
| Badila vs. Ledong2 Flower upDEGs KEGG enrichment | | | | | | | |
| ID | Description | GeneRatio | BgRatio | pvalue | p.adjust | qvalue | Count |
| ko00194 | Photosynthesis proteins | 0.0150342 | 0.005687446 | 1.05E-24 | 3.74E-22 | 2.87E-22 | 101 |
| ko00195 | Photosynthesis | 0.0125037 | 0.00444132 | 2.61E-23 | 4.65E-21 | 3.57E-21 | 84 |
| ko00710 | Carbon fixation in photosynthetic organisms | 0.0162251 | 0.008083842 | 8.41E-15 | 9.98E-13 | 7.67E-13 | 109 |
| ko04371 | Apelin signaling pathway | 0.0151831 | 0.007860178 | 1.08E-12 | 9.61E-11 | 7.39E-11 | 102 |
| ko05410 | Hypertrophic cardiomyopathy (HCM) | 0.008187 | 0.00361057 | 1.37E-10 | 9.75E-09 | 7.50E-09 | 55 |
| ko04931 | Insulin resistance | 0.013248 | 0.007125284 | 3.14E-10 | 1.86E-08 | 1.43E-08 | 89 |
| ko04140 | Autophagy − animal | 0.0166716 | 0.009777295 | 8.66E-10 | 4.40E-08 | 3.39E-08 | 112 |
| ko04910 | Insulin signaling pathway | 0.018309 | 0.011151229 | 1.97E-09 | 8.77E-08 | 6.74E-08 | 123 |
| ko00630 | Glyoxylate and dicarboxylate metabolism | 0.0130991 | 0.007444803 | 9.56E-09 | 3.78E-07 | 2.91E-07 | 88 |
| ko04920 | Adipocytokine signaling pathway | 0.0092289 | 0.004920599 | 9.83E-08 | 3.50E-06 | 2.69E-06 | 62 |
|  |  |  |  |  |  |  |  |
| Badila vs. Ledong2 Flower downDEGs KEGG enrichment | | | | | | | |
| ID | Description | GeneRatio | BgRatio | pvalue | p.adjust | qvalue | Count |
| ko05322 | Systemic lupus erythematosus | 0.012946 | 0.004473272 | 6.99E-25 | 2.53E-22 | 2.29E-22 | 86 |
| ko03030 | DNA replication | 0.0127954 | 0.006646004 | 1.06E-10 | 1.92E-08 | 1.73E-08 | 85 |
| ko03430 | Mismatch repair | 0.0100858 | 0.005272071 | 1.29E-08 | 1.56E-06 | 1.41E-06 | 67 |
| ko05203 | Viral carcinogenesis | 0.0185157 | 0.011758315 | 3.38E-08 | 3.06E-06 | 2.77E-06 | 123 |
| ko05034 | Alcoholism | 0.0141502 | 0.008595073 | 1.35E-07 | 9.78E-06 | 8.85E-06 | 94 |
| ko00062 | Fatty acid elongation | 0.0082794 | 0.004281561 | 1.62E-07 | 9.78E-06 | 8.85E-06 | 55 |
| ko04540 | Gap junction | 0.0039139 | 0.001501741 | 3.24E-07 | 1.68E-05 | 1.52E-05 | 26 |
| ko05130 | Pathogenic Escherichia coli infection | 0.0069246 | 0.003578618 | 1.57E-06 | 7.12E-05 | 6.44E-05 | 46 |
| ko03440 | Homologous recombination | 0.0117417 | 0.007253091 | 3.12E-06 | 0.000121228 | 0.00011 | 78 |
| ko04972 | Pancreatic secretion | 0.0054192 | 0.002620059 | 3.35E-06 | 0.000121228 | 0.00011 | 36 |
