# Supplementary material for: Differential gene expression and phenotypic variation across tissues between Saccharum officinarum and Saccharum spontaneum
Source: Front Plant Sci. 2025 Oct 31;16:1696921. doi: 10.3389/fpls.2025.1696921 (PMC12617224; doi:10.3389/fpls.2025.1696921)
Supplement: Supplementary Figure 1 — Gene expression (log2TPM) in four tissues including root, stem, leaf, and flower between Badila and Ledong2. TPM: transcripts per million. [file DataSheet1.zip › Supplement information-0901/Supplementary Table S4 GO enrichment of DEGs in root.docx]

**Table S4** Gene ontology (GO) enrichment of differentially expressed genes (DEGs) in root tissues from Badila and Ledong2.

| **GO.ID** | **Term Description** | **Annotated Genes** | **Significant Genes** | **Expected value** | **Enrichment** | **p value** | **GO term** |
| --- | --- | --- | --- | --- | --- | --- | --- |
| **Upregulation DEGs in root comparison of Badila with Ledong2** | | | | | | | |
| GO:0009737 | response to abscisic acid | 1613 | 353 | 284.7 | 8.200659451 | 6.30E-09 | BP |
| GO:0009651 | response to salt stress | 1633 | 356 | 288.23 | 6.420216403 | 3.80E-07 | BP |
| GO:2000030 | regulation of response to red or far red light | 105 | 30 | 18.53 | 5.455931956 | 3.50E-06 | BP |
| GO:0046256 | 2,4,6-trinitrotoluene catabolic process | 20 | 13 | 3.53 | 5.443697499 | 3.60E-06 | BP |
| GO:0009635 | response to herbicide | 54 | 22 | 9.53 | 5.30980392 | 4.90E-06 | BP |
| GO:0009835 | fruit ripening | 30 | 16 | 5.3 | 5.031517051 | 9.30E-06 | BP |
| GO:0006805 | xenobiotic metabolic process | 22 | 13 | 3.88 | 4.795880017 | 1.60E-05 | BP |
| GO:0007080 | mitotic metaphase plate congression | 65 | 22 | 11.47 | 4.795880017 | 1.60E-05 | BP |
| GO:0007018 | microtubule-based movement | 239 | 69 | 42.18 | 4.657577319 | 2.20E-05 | BP |
| GO:0072530 | purine-containing compound transmembrane transport | 47 | 16 | 8.3 | 4.657577319 | 2.20E-05 | BP |
| GO:0031012 | extracellular matrix | 56 | 31 | 9.63 | 9.958607315 | 1.10E-10 | CC |
| GO:0000325 | plant-type vacuole | 556 | 123 | 95.58 | 8.657577319 | 2.20E-09 | CC |
| GO:0005871 | kinesin complex | 175 | 53 | 30.08 | 4.853871964 | 1.40E-05 | CC |
| GO:0033597 | mitotic checkpoint complex | 17 | 11 | 2.92 | 4.769551079 | 1.70E-05 | CC |
| GO:0001669 | acrosomal vesicle | 11 | 8 | 1.89 | 4.119186408 | 7.60E-05 | CC |
| GO:0002177 | manchette | 11 | 8 | 1.89 | 4.119186408 | 7.60E-05 | CC |
| GO:0098687 | chromosomal region | 273 | 71 | 46.93 | 3.292429824 | 0.00051 | CC |
| GO:0000792 | heterochromatin | 54 | 16 | 9.28 | 2.812479279 | 0.00154 | CC |
| GO:0005743 | mitochondrial inner membrane | 436 | 79 | 74.95 | 2.591760035 | 0.00256 | CC |
| GO:0035371 | microtubule plus-end | 39 | 14 | 6.7 | 2.404503778 | 0.00394 | CC |
| GO:0004497 | monooxygenase activity | 480 | 123 | 85.54 | 7.27572413 | 5.30E-08 | MF |
| GO:0043295 | glutathione binding | 53 | 25 | 9.44 | 6.060480747 | 8.70E-07 | MF |
| GO:0004364 | glutathione transferase activity | 78 | 32 | 13.9 | 5.853871964 | 1.40E-06 | MF |
| GO:0004564 | beta-fructofuranosidase activity | 32 | 12 | 5.7 | 5.15490196 | 7.00E-06 | MF |
| GO:0000295 | adenine nucleotide transmembrane transporter activity | 54 | 19 | 9.62 | 4.050609993 | 8.90E-05 | MF |
| GO:0005346 | purine ribonucleotide transmembrane transporter activity | 54 | 19 | 9.62 | 4.050609993 | 8.90E-05 | MF |
| GO:0004888 | transmembrane signaling receptor activity | 432 | 86 | 76.98 | 3.537602002 | 0.00029 | MF |
| GO:0003680 | AT DNA binding | 24 | 12 | 4.28 | 3.494850022 | 0.00032 | MF |
| GO:0000247 | C-8 sterol isomerase activity | 10 | 7 | 1.78 | 3.387216143 | 0.00041 | MF |
| GO:0050403 | trans-zeatin O-beta-D-glucosyltransferase activity | 22 | 11 | 3.92 | 3.236572006 | 0.00058 | MF |
| **Downregulation DEGs in root comparison of Badila with Ledong2** | | | | | | | |
| GO:0042866 | pyruvate biosynthetic process | 99 | 42 | 15.12 | 10.09151498 | 8.1E-11 | BP |
| GO:0019359 | nicotinamide nucleotide biosynthetic process | 122 | 47 | 18.63 | 8.886056648 | 1.3E-09 | BP |
| GO:0006754 | ATP biosynthetic process | 149 | 45 | 22.75 | 7.744727495 | 1.8E-08 | BP |
| GO:0051186 | cofactor metabolic process | 1084 | 212 | 165.52 | 7.387216143 | 4.1E-08 | BP |
| GO:0009168 | purine ribonucleoside monophosphate biosynthetic process | 152 | 50 | 23.21 | 7.346787486 | 4.5E-08 | BP |
| GO:0006096 | glycolytic process | 107 | 40 | 16.34 | 6.958607315 | 1.1E-07 | BP |
| GO:0009166 | nucleotide catabolic process | 119 | 41 | 18.17 | 6.769551079 | 1.7E-07 | BP |
| GO:0015837 | amine transport | 14 | 11 | 2.14 | 6.619788758 | 2.4E-07 | BP |
| GO:0006094 | gluconeogenesis | 47 | 21 | 7.18 | 5.823908741 | 1.5E-06 | BP |
| GO:2000280 | regulation of root development | 233 | 48 | 35.58 | 5.744727495 | 1.8E-06 | BP |
| GO:0009505 | plant-type cell wall | 658 | 161 | 101.47 | 9.397940009 | 4E-10 | CC |
| GO:0009506 | plasmodesma | 2059 | 398 | 317.53 | 6.387216143 | 4.1E-07 | CC |
| GO:0034455 | t-UTP complex | 12 | 9 | 1.85 | 5.161150909 | 6.9E-06 | CC |
| GO:0071013 | catalytic step 2 spliceosome | 143 | 33 | 22.05 | 3.721246399 | 0.00019 | CC |
| GO:0005829 | cytosol | 5117 | 870 | 789.12 | 3.677780705 | 0.00021 | CC |
| GO:0005740 | mitochondrial envelope | 588 | 106 | 90.68 | 3.443697499 | 0.00036 | CC |
| GO:0005682 | U5 snRNP | 33 | 13 | 5.09 | 3.13076828 | 0.00074 | CC |
| GO:0005764 | lysosome | 182 | 38 | 28.07 | 2.93930216 | 0.00115 | CC |
| GO:0009570 | chloroplast stroma | 1676 | 292 | 258.46 | 2.612610174 | 0.00244 | CC |
| GO:0045178 | basal part of cell | 16 | 8 | 2.47 | 2.607303047 | 0.00247 | CC |
| GO:0004069 | L-aspartate:2-oxoglutarate aminotransferase activity | 11 | 10 | 1.72 | 7.075720714 | 8.4E-08 | MF |
| GO:0005507 | copper ion binding | 269 | 73 | 42.17 | 6.004364805 | 9.9E-07 | MF |
| GO:0047262 | polygalacturonate 4-alpha-galacturonosyltransferase activity | 56 | 21 | 8.78 | 4.22184875 | 0.00006 | MF |
| GO:0008889 | glycerophosphodiester phosphodiesterase activity | 24 | 12 | 3.76 | 4.040958608 | 0.000091 | MF |
| GO:0004611 | phosphoenolpyruvate carboxykinase activity | 28 | 6 | 4.39 | 4.022276395 | 0.000095 | MF |
| GO:0016208 | AMP binding | 18 | 10 | 2.82 | 3.958607315 | 0.00011 | MF |
| GO:0016231 | beta-N-acetylglucosaminidase activity | 10 | 7 | 1.57 | 3.744727495 | 0.00018 | MF |
| GO:0004332 | fructose-bisphosphate aldolase activity | 13 | 8 | 2.04 | 3.657577319 | 0.00022 | MF |
| GO:0015250 | water channel activity | 105 | 31 | 16.46 | 3.602059991 | 0.00025 | MF |
| GO:0015210 | uracil transmembrane transporter activity | 15 | 8 | 2.35 | 3.086186148 | 0.00082 | MF |

BP: Biological process; CC: cellular component; MF: Molecular function.
