# Supplementary material for: Differential gene expression and phenotypic variation across tissues between Saccharum officinarum and Saccharum spontaneum
Source: Front Plant Sci. 2025 Oct 31;16:1696921. doi: 10.3389/fpls.2025.1696921 (PMC12617224; doi:10.3389/fpls.2025.1696921)
Supplement: Supplementary Figure 1 — Gene expression (log2TPM) in four tissues including root, stem, leaf, and flower between Badila and Ledong2. TPM: transcripts per million. [file DataSheet1.zip › Supplement information-0901/Supplementary Table S5 GO enrichment of DEGs in stem.docx]

**Table S5** Gene ontology (GO) enrichment of differentially expressed genes (DEGs) in stem tissues from Badila and Ledong2.

| **GO.ID** | **Term Description** | **Annotated Genes** | **Significant Genes** | **Expected value** | **Enrichment** | **p value** | **GO term** |
| --- | --- | --- | --- | --- | --- | --- | --- |
| **Upregulation DEGs in stem comparison of Badila with Ledong2** | | | | | | | |
| GO:0007018 | microtubule-based movement | 239 | 165 | 61.95 | 30 | 1.00E-30 | BP |
| GO:0009664 | plant-type cell wall organization | 373 | 167 | 96.69 | 13.09151498 | 8.10E-14 | BP |
| GO:0006268 | DNA unwinding involved in DNA replication | 37 | 31 | 9.59 | 12.58502665 | 2.60E-13 | BP |
| GO:0007140 | male meiotic nuclear division | 88 | 47 | 22.81 | 10.88605665 | 1.30E-11 | BP |
| GO:0001558 | regulation of cell growth | 294 | 102 | 76.21 | 10.31875876 | 4.80E-11 | BP |
| GO:0007129 | synapsis | 93 | 54 | 24.11 | 9.283996656 | 5.20E-10 | BP |
| GO:0051302 | regulation of cell division | 295 | 103 | 76.47 | 9.013228266 | 9.70E-10 | BP |
| GO:0000712 | resolution of meiotic recombination intermediates | 51 | 34 | 13.22 | 8.920818754 | 1.20E-09 | BP |
| GO:0043622 | cortical microtubule organization | 113 | 59 | 29.29 | 8.657577319 | 2.20E-09 | BP |
| GO:0016572 | histone phosphorylation | 22 | 19 | 5.7 | 8.327902142 | 4.70E-09 | BP |
| GO:0005871 | kinesin complex | 175 | 143 | 46.37 | 30 | 1.00E-30 | CC |
| GO:0009505 | plant-type cell wall | 658 | 268 | 174.34 | 12.49485002 | 3.20E-13 | CC |
| GO:0005875 | microtubule associated complex | 244 | 190 | 64.65 | 12.14874165 | 7.10E-13 | CC |
| GO:0009535 | chloroplast thylakoid membrane | 615 | 240 | 162.95 | 10.06550155 | 8.60E-11 | CC |
| GO:0005874 | microtubule | 285 | 181 | 75.51 | 9.677780705 | 2.10E-10 | CC |
| GO:0046658 | anchored component of plasma membrane | 306 | 131 | 81.08 | 9.37675071 | 4.20E-10 | CC |
| GO:0035371 | microtubule plus-end | 39 | 29 | 10.33 | 9.22184875 | 6.00E-10 | CC |
| GO:0042555 | MCM complex | 24 | 20 | 6.36 | 8.022276395 | 9.50E-09 | CC |
| GO:0005876 | spindle microtubule | 62 | 45 | 16.43 | 8.013228266 | 9.70E-09 | CC |
| GO:0009524 | phragmoplast | 205 | 90 | 54.32 | 7.283996656 | 5.20E-08 | CC |
| GO:0003777 | microtubule motor activity | 175 | 143 | 44.1 | 30 | 1.00E-30 | MF |
| GO:0008574 | ATP-dependent microtubule motor activity, plus-end-directed | 49 | 41 | 12.35 | 16.92081875 | 1.20E-17 | MF |
| GO:0008017 | microtubule binding | 223 | 112 | 56.2 | 13.09151498 | 8.10E-14 | MF |
| GO:0016887 | ATPase activity | 1214 | 356 | 305.94 | 12.20065945 | 6.30E-13 | MF |
| GO:0003677 | DNA binding | 2509 | 718 | 632.29 | 12.1739252 | 6.70E-13 | MF |
| GO:0008569 | ATP-dependent microtubule motor activity, minus-end-directed | 16 | 16 | 4.03 | 9.585026652 | 2.60E-10 | MF |
| GO:0033612 | receptor serine/threonine kinase binding | 111 | 47 | 27.97 | 7.397940009 | 4.00E-08 | MF |
| GO:0003682 | chromatin binding | 394 | 136 | 99.29 | 6.698970004 | 2.00E-07 | MF |
| GO:0004675 | transmembrane receptor protein serine/threonine kinase activity | 344 | 129 | 86.69 | 6.585026652 | 2.60E-07 | MF |
| GO:0030527 | structural constituent of chromatin | 11 | 11 | 2.77 | 6.585026652 | 2.60E-07 | MF |
| **Downregulation DEGs in stem comparison of Badila with Ledong2** | | | | | | | |
| GO:0010200 | response to chitin | 286 | 113 | 58.31 | 10.82390874 | 1.50E-11 | BP |
| GO:0009611 | response to wounding | 725 | 207 | 147.82 | 9.552841969 | 2.80E-10 | BP |
| GO:2000022 | regulation of jasmonic acid mediated signaling pathway | 84 | 42 | 17.13 | 8.853871964 | 1.40E-09 | BP |
| GO:1901002 | positive regulation of response to salt stress | 92 | 44 | 18.76 | 8.455931956 | 3.50E-09 | BP |
| GO:0010043 | response to zinc ion | 87 | 42 | 17.74 | 8.259637311 | 5.50E-09 | BP |
| GO:1900057 | positive regulation of leaf senescence | 97 | 44 | 19.78 | 7.585026652 | 2.60E-08 | BP |
| GO:0009414 | response to water deprivation | 954 | 272 | 194.51 | 7.522878745 | 3.00E-08 | BP |
| GO:0051704 | multi-organism process | 3704 | 850 | 755.19 | 7.494850022 | 3.20E-08 | BP |
| GO:0001666 | response to hypoxia | 188 | 66 | 38.33 | 6.920818754 | 1.20E-07 | BP |
| GO:0035556 | intracellular signal transduction | 1313 | 370 | 267.7 | 6.853871964 | 1.40E-07 | BP |
| GO:0005777 | peroxisome | 549 | 168 | 109.86 | 6.337242168 | 4.60E-07 | CC |
| GO:0030018 | Z disc | 22 | 13 | 4.4 | 4.187086643 | 6.50E-05 | CC |
| GO:0005886 | plasma membrane | 5941 | 1285 | 1188.87 | 3.657577319 | 0.00022 | CC |
| GO:0005778 | peroxisomal membrane | 84 | 31 | 16.81 | 3.602059991 | 0.00025 | CC |
| GO:0009898 | cytoplasmic side of plasma membrane | 53 | 18 | 10.61 | 3.508638306 | 0.00031 | CC |
| GO:0000151 | ubiquitin ligase complex | 471 | 109 | 94.25 | 3.37675071 | 0.00042 | CC |
| GO:0034455 | t-UTP complex | 12 | 8 | 2.4 | 3.236572006 | 0.00058 | CC |
| GO:0000327 | lytic vacuole within protein storage vacuole | 10 | 7 | 2 | 3.060480747 | 0.00087 | CC |
| GO:0016460 | myosin II complex | 10 | 7 | 2 | 3.060480747 | 0.00087 | CC |
| GO:0120025 | plasma membrane bounded cell projection | 306 | 63 | 61.23 | 3.036212173 | 0.00092 | CC |
| GO:0004712 | protein serine/threonine/tyrosine kinase activity | 126 | 48 | 26.28 | 7.091514981 | 8.10E-08 | MF |
| GO:0016165 | linoleate 13S-lipoxygenase activity | 31 | 20 | 6.47 | 6.744727495 | 1.80E-07 | MF |
| GO:0004672 | protein kinase activity | 1692 | 444 | 352.94 | 6.13076828 | 7.40E-07 | MF |
| GO:0033293 | monocarboxylic acid binding | 61 | 28 | 12.72 | 5.958607315 | 1.10E-06 | MF |
| GO:0005516 | calmodulin binding | 365 | 113 | 76.14 | 5.48148606 | 3.30E-06 | MF |
| GO:0004709 | MAP kinase kinase kinase activity | 41 | 22 | 8.55 | 5.420216403 | 3.80E-06 | MF |
| GO:0015179 | L-amino acid transmembrane transporter activity | 72 | 28 | 15.02 | 5.193820026 | 6.40E-06 | MF |
| GO:0097159 | organic cyclic compound binding | 5784 | 1172 | 1206.5 | 5.15490196 | 7.00E-06 | MF |
| GO:0016645 | oxidoreductase activity, acting on the CH-NH group of donors | 103 | 27 | 21.49 | 4.769551079 | 1.70E-05 | MF |
| GO:0043565 | sequence-specific DNA binding | 1511 | 354 | 315.18 | 4.619788758 | 2.40E-05 | MF |

BP: Biological process; CC: cellular component; MF: Molecular function.
