# Supplementary material for: Differential gene expression and phenotypic variation across tissues between Saccharum officinarum and Saccharum spontaneum
Source: Front Plant Sci. 2025 Oct 31;16:1696921. doi: 10.3389/fpls.2025.1696921 (PMC12617224; doi:10.3389/fpls.2025.1696921)
Supplement: Supplementary Figure 1 — Gene expression (log2TPM) in four tissues including root, stem, leaf, and flower between Badila and Ledong2. TPM: transcripts per million. [file DataSheet1.zip › Supplement information-0901/Supplementary Table S6 GO enrichment of DEGs in leaf.docx]

**Table** S6 Gene ontology (GO) enrichment of differentially expressed genes (DEGs) in leaf tissues from Badila and Ledong2.

| **GO. ID** | **Term Description** | **Annotated Genes** | **Significant Genes** | **Expected value** | **Enrichment** | **p value** | **GO term** |
| --- | --- | --- | --- | --- | --- | --- | --- |
| **Upregulation DEGs in leaf comparison of Badila with Ledong2** | | | | | | | |
| GO:0048871 | multicellular organismal homeostasis | 36 | 18 | 6.02 | 10.1079054 | 7.80E-11 | BP |
| GO:0009635 | response to herbicide | 54 | 24 | 9.03 | 7.142667504 | 7.20E-08 | BP |
| GO:0009753 | response to jasmonic acid | 540 | 129 | 90.28 | 6.958607315 | 1.10E-07 | BP |
| GO:0009773 | photosynthetic electron transport in photosystem I | 38 | 20 | 6.35 | 6.366531544 | 4.30E-07 | BP |
| GO:0035556 | intracellular signal transduction | 1313 | 280 | 219.51 | 5.008773924 | 9.80E-06 | BP |
| GO:0009767 | photosynthetic electron transport chain | 117 | 42 | 19.56 | 4.920818754 | 1.20E-05 | BP |
| GO:0006168 | adenine salvage | 12 | 9 | 2.01 | 4.853871964 | 1.40E-05 | BP |
| GO:0019499 | cyanide metabolic process | 10 | 8 | 1.67 | 4.698970004 | 2.00E-05 | BP |
| GO:0051410 | detoxification of nitrogen compound | 10 | 8 | 1.67 | 4.698970004 | 2.00E-05 | BP |
| GO:1901575 | organic substance catabolic process | 2712 | 484 | 453.39 | 4.677780705 | 2.10E-05 | BP |
| GO:0009570 | chloroplast stroma | 1676 | 469 | 281.43 | 29.22184875 | 6.00E-30 | CC |
| GO:0009535 | chloroplast thylakoid membrane | 615 | 194 | 103.27 | 18.61978876 | 2.40E-19 | CC |
| GO:0031977 | thylakoid lumen | 163 | 72 | 27.37 | 11.00436481 | 9.90E-12 | CC |
| GO:0010598 | NAD(P)H dehydrogenase complex (plastoquinone) | 43 | 26 | 7.22 | 9.853871964 | 1.40E-10 | CC |
| GO:0009941 | chloroplast envelope | 1351 | 315 | 226.86 | 9.356547324 | 4.40E-10 | CC |
| GO:0005622 | intracellular | 24606 | 4304 | 4131.77 | 6.387216143 | 4.10E-07 | CC |
| GO:0009543 | chloroplast thylakoid lumen | 42 | 20 | 7.05 | 5.455931956 | 3.50E-06 | CC |
| GO:0009507 | chloroplast | 5020 | 1146 | 842.94 | 4.721246399 | 1.90E-05 | CC |
| GO:0009533 | chloroplast stromal thylakoid | 18 | 11 | 3.02 | 4.537602002 | 2.90E-05 | CC |
| GO:0005737 | cytoplasm | 18740 | 3353 | 3146.77 | 3.568636236 | 0.00027 | CC |
| GO:0043295 | glutathione binding | 53 | 27 | 8.95 | 7.886056648 | 1.30E-08 | MF |
| GO:0004364 | glutathione transferase activity | 78 | 33 | 13.17 | 6.958607315 | 1.10E-07 | MF |
| GO:0016671 | oxidoreductase activity, acting on a sulfur group of donors … | 109 | 38 | 18.41 | 5.37675071 | 4.20E-06 | MF |
| GO:0003999 | adenine phosphoribosyltransferase activity | 12 | 9 | 2.03 | 4.823908741 | 1.50E-05 | MF |
| GO:0004564 | beta-fructofuranosidase activity | 32 | 12 | 5.4 | 4.173925197 | 6.70E-05 | MF |
| GO:0005509 | calcium ion binding | 259 | 68 | 43.74 | 4.055517328 | 8.80E-05 | MF |
| GO:0016846 | carbon-sulfur lyase activity | 60 | 18 | 10.13 | 4.008773924 | 9.80E-05 | MF |
| GO:0050308 | sugar-phosphatase activity | 25 | 10 | 4.22 | 3.677780705 | 0.00021 | MF |
| GO:0004845 | uracil phosphoribosyltransferase activity | 13 | 8 | 2.2 | 3.420216403 | 0.00038 | MF |
| GO:0004674 | protein serine/threonine kinase activity | 1402 | 238 | 236.78 | 3.30980392 | 0.00049 | MF |
| **Downregulation DEGs in leaf comparison of Badila with Ledong2** | | | | | | | |
| GO:0034440 | lipid oxidation | 164 | 31 | 26.97 | 7.920818754 | 0.000000012 | BP |
| GO:0032259 | methylation | 828 | 177 | 136.17 | 7.070581074 | 0.000000085 | BP |
| GO:0051649 | establishment of localization in cell | 1608 | 296 | 264.45 | 5.142667504 | 0.0000072 | BP |
| GO:0033169 | histone H3-K9 demethylation | 25 | 14 | 4.11 | 5.124938737 | 0.0000075 | BP |
| GO:0009698 | phenylpropanoid metabolic process | 305 | 55 | 50.16 | 4.619788758 | 0.000024 | BP |
| GO:0051784 | negative regulation of nuclear division | 82 | 21 | 13.49 | 4.070581074 | 0.000085 | BP |
| GO:0002832 | negative regulation of response to biotic stimulus | 125 | 28 | 20.56 | 3.920818754 | 0.00012 | BP |
| GO:0005982 | starch metabolic process | 188 | 50 | 30.92 | 3.886056648 | 0.00013 | BP |
| GO:0043547 | positive regulation of GTPase activity | 111 | 28 | 18.25 | 3.823908741 | 0.00015 | BP |
| GO:0009616 | virus induced gene silencing | 53 | 18 | 8.72 | 3.769551079 | 0.00017 | BP |
| GO:0009506 | plasmodesma | 2059 | 419 | 344.29 | 5.356547324 | 0.0000044 | CC |
| GO:0005829 | cytosol | 5117 | 987 | 855.63 | 5.251811973 | 0.0000056 | CC |
| GO:0005802 | trans-Golgi network | 700 | 163 | 117.05 | 4.214670165 | 0.000061 | CC |
| GO:0005832 | chaperonin-containing T-complex | 32 | 15 | 5.35 | 4.15490196 | 0.00007 | CC |
| GO:0022625 | cytosolic large ribosomal subunit | 228 | 61 | 38.12 | 4.070581074 | 0.000085 | CC |
| GO:0005655 | nucleolar ribonuclease P complex | 17 | 10 | 2.84 | 4 | 0.0001 | CC |
| GO:0009706 | chloroplast inner membrane | 155 | 43 | 25.92 | 3.853871964 | 0.00014 | CC |
| GO:0005950 | anthranilate synthase complex | 12 | 8 | 2.01 | 3.795880017 | 0.00016 | CC |
| GO:0005886 | plasma membrane | 5941 | 1069 | 993.41 | 3.552841969 | 0.00028 | CC |
| GO:0009897 | external side of plasma membrane | 42 | 16 | 7.02 | 3.124938737 | 0.00075 | CC |
| GO:0016207 | 4-coumarate-CoA ligase activity | 22 | 15 | 3.67 | 6.958607315 | 0.00000011 | MF |
| GO:0008757 | S-adenosylmethionine-dependent methyltransferase activity | 437 | 112 | 72.97 | 5.657577319 | 0.0000022 | MF |
| GO:0016410 | N-acyltransferase activity | 103 | 24 | 17.2 | 5.585026652 | 0.0000026 | MF |
| GO:0016165 | linoleate 13S-lipoxygenase activity | 31 | 15 | 5.18 | 4.366531544 | 0.000043 | MF |
| GO:0047262 | polygalacturonate 4-alpha-galacturonosyltransferase activity | 56 | 22 | 9.35 | 4.327902142 | 0.000047 | MF |
| GO:0016874 | ligase activity | 425 | 102 | 70.97 | 4.318758763 | 0.000048 | MF |
| GO:0004049 | anthranilate synthase activity | 12 | 8 | 2 | 3.795880017 | 0.00016 | MF |
| GO:0016538 | cyclin-dependent protein serine/threonine kinase regulator … | 47 | 17 | 7.85 | 3.698970004 | 0.0002 | MF |
| GO:0003714 | transcription corepressor activity | 78 | 26 | 13.03 | 3.602059991 | 0.00025 | MF |
| GO:0016231 | beta-N-acetylglucosaminidase activity | 10 | 7 | 1.67 | 3.568636236 | 0.00027 | MF |

BP: Biological process; CC: cellular component; MF: Molecular function.
