# Supplementary material for: Differential gene expression and phenotypic variation across tissues between Saccharum officinarum and Saccharum spontaneum
Source: Front Plant Sci. 2025 Oct 31;16:1696921. doi: 10.3389/fpls.2025.1696921 (PMC12617224; doi:10.3389/fpls.2025.1696921)
Supplement: Supplementary Figure 1 — Gene expression (log2TPM) in four tissues including root, stem, leaf, and flower between Badila and Ledong2. TPM: transcripts per million. [file DataSheet1.zip › Supplement information-0901/Supplementary Table S7 GO enrichment of DEGs in flower.docx]

**Table S7** Gene ontology (GO) enrichment of differentially expressed genes (DEGs) in flowering tissues from Badila and Ledong2.

| **GO. ID** | **Term Description** | **Annotated Genes** | **Significant Genes** | **Expected value** | **Enrichment** | **p value** | **GO term** |
| --- | --- | --- | --- | --- | --- | --- | --- |
| **Upregulation DEGs in flowing comparison of Badila with Ledong2** | | | | | | | |
| GO:0009737 | response to abscisic acid | 1613 | 524 | 362.15 | 20.74472749 | 1.80E-21 | BP |
| GO:0009414 | response to water deprivation | 954 | 330 | 214.19 | 14.1079054 | 7.80E-15 | BP |
| GO:0080167 | response to karrikin | 350 | 134 | 78.58 | 10.85387196 | 1.40E-11 | BP |
| GO:0009753 | response to jasmonic acid | 540 | 191 | 121.24 | 10.56863624 | 2.70E-11 | BP |
| GO:0009835 | fruit ripening | 30 | 17 | 6.74 | 10.38721614 | 4.10E-11 | BP |
| GO:0009409 | response to cold | 1052 | 316 | 236.19 | 10.27572413 | 5.30E-11 | BP |
| GO:0009704 | de-etiolation | 41 | 29 | 9.21 | 10.20760831 | 6.20E-11 | BP |
| GO:1900057 | positive regulation of leaf senescence | 97 | 49 | 21.78 | 8.886056648 | 1.30E-09 | BP |
| GO:0009644 | response to high light intensity | 220 | 96 | 49.39 | 8.698970004 | 2.00E-09 | BP |
| GO:0071483 | cellular response to blue light | 61 | 26 | 13.7 | 8.408935393 | 3.90E-09 | BP |
| GO:0009535 | chloroplast thylakoid membrane | 615 | 294 | 135.85 | 30 | 1.00E-30 | CC |
| GO:0010287 | plastoglobule | 122 | 72 | 26.95 | 17.92081875 | 1.20E-18 | CC |
| GO:0009941 | chloroplast envelope | 1351 | 438 | 298.43 | 16.7212464 | 1.90E-17 | CC |
| GO:0009570 | chloroplast stroma | 1676 | 559 | 370.22 | 15.7212464 | 1.90E-16 | CC |
| GO:0009507 | chloroplast | 5020 | 1525 | 1108.89 | 10.82390874 | 1.50E-11 | CC |
| GO:0031977 | thylakoid lumen | 163 | 69 | 36.01 | 9.008773924 | 9.80E-10 | CC |
| GO:0005811 | lipid droplet | 74 | 39 | 16.35 | 8.075720714 | 8.40E-09 | CC |
| GO:0010598 | NAD(P)H dehydrogenase complex (plastoquinone) | 43 | 27 | 9.5 | 7.958607315 | 1.10E-08 | CC |
| GO:0009533 | chloroplast stromal thylakoid | 18 | 14 | 3.98 | 6.102372909 | 7.90E-07 | CC |
| GO:0000407 | phagophore assembly site | 43 | 20 | 9.5 | 5.619788758 | 2.40E-06 | CC |
| GO:0005509 | calcium ion binding | 259 | 93 | 58.15 | 6.27572413 | 5.30E-07 | MF |
| GO:0004144 | diacylglycerol O-acyltransferase activity | 36 | 22 | 8.08 | 6.167491087 | 6.80E-07 | MF |
| GO:0016161 | beta-amylase activity | 19 | 14 | 4.27 | 5.537602002 | 2.90E-06 | MF |
| GO:0004709 | MAP kinase kinase kinase activity | 41 | 23 | 9.2 | 5.508638306 | 3.10E-06 | MF |
| GO:0022857 | transmembrane transporter activity | 2196 | 638 | 493.02 | 5.431798276 | 3.70E-06 | MF |
| GO:0016151 | nickel cation binding | 22 | 15 | 4.94 | 5.22184875 | 6.00E-06 | MF |
| GO:0042132 | fructose 1,6-bisphosphate 1-phosphatase activity | 10 | 9 | 2.25 | 4.920818754 | 1.20E-05 | MF |
| GO:0042887 | amide transmembrane transporter activity | 99 | 26 | 22.23 | 4.920818754 | 1.20E-05 | MF |
| GO:0008422 | beta-glucosidase activity | 137 | 49 | 30.76 | 4.886056648 | 1.30E-05 | MF |
| GO:0016810 | hydrolase activity, acting on carbon-nitrogen… | 220 | 58 | 49.39 | 4.853871964 | 1.40E-05 | MF |
| **Downregulation DEGs in flowing comparison of Badila with Ledong2** | | | | | | | |
| GO:0007018 | microtubule-based movement | 239 | 135 | 52.1 | 30 | 1E-30 | BP |
| GO:0009834 | plant-type secondary cell wall biogenesis | 219 | 100 | 47.74 | 18.82390874 | 1.5E-19 | BP |
| GO:0007140 | male meiotic nuclear division | 88 | 41 | 19.18 | 11.25181197 | 5.6E-12 | BP |
| GO:0009699 | phenylpropanoid biosynthetic process | 256 | 103 | 55.8 | 9.26760624 | 5.4E-10 | BP |
| GO:0052325 | cell wall pectin biosynthetic process | 47 | 35 | 10.25 | 9.15490196 | 7E-10 | BP |
| GO:0048367 | shoot system development | 2482 | 574 | 541.03 | 9.036212173 | 9.2E-10 | BP |
| GO:0010143 | cutin biosynthetic process | 77 | 40 | 16.78 | 8.769551079 | 1.7E-09 | BP |
| GO:0007178 | transmembrane receptor protein serine/threonine kinase signal … | 376 | 125 | 81.96 | 8.698970004 | 2E-09 | BP |
| GO:0010215 | cellulose microfibril organization | 17 | 15 | 3.71 | 8 | 1E-08 | BP |
| GO:0010338 | leaf formation | 17 | 15 | 3.71 | 8 | 1E-08 | BP |
| GO:0005871 | kinesin complex | 175 | 116 | 39.67 | 30 | 1E-30 | CC |
| GO:0009505 | plant-type cell wall | 658 | 259 | 149.17 | 21.33724217 | 4.6E-22 | CC |
| GO:0005802 | trans-Golgi network | 700 | 266 | 158.7 | 18.76955108 | 1.7E-19 | CC |
| GO:0009506 | plasmodesma | 2059 | 625 | 466.79 | 16.48148606 | 3.3E-17 | CC |
| GO:0005794 | Golgi apparatus | 2025 | 685 | 459.08 | 12.45593196 | 3.5E-13 | CC |
| GO:0005768 | endosome | 1078 | 328 | 244.39 | 12.1426675 | 7.2E-13 | CC |
| GO:0005856 | cytoskeleton | 980 | 438 | 222.17 | 12.08092191 | 8.3E-13 | CC |
| GO:0005874 | microtubule | 285 | 167 | 64.61 | 12.06048075 | 8.7E-13 | CC |
| GO:0009524 | phragmoplast | 205 | 91 | 46.48 | 11.37675071 | 4.2E-12 | CC |
| GO:0046658 | anchored component of plasma membrane | 306 | 117 | 69.37 | 9.26760624 | 5.4E-10 | CC |
| GO:0003777 | microtubule motor activity | 175 | 116 | 37.49 | 20.21467016 | 6.1E-21 | MF |
| GO:0008017 | microtubule binding | 223 | 114 | 47.78 | 17.30103 | 5E-18 | MF |
| GO:0008574 | ATP-dependent microtubule motor activity, plus-end-directed | 49 | 33 | 10.5 | 11.20065945 | 6.3E-12 | MF |
| GO:0016887 | ATPase activity | 1214 | 330 | 260.09 | 10.38721614 | 4.1E-11 | MF |
| GO:0004675 | transmembrane receptor protein serine/threonine kinase activity | 344 | 123 | 73.7 | 9.229147988 | 5.9E-10 | MF |
| GO:0042285 | xylosyltransferase activity | 86 | 32 | 18.42 | 8.259637311 | 5.5E-09 | MF |
| GO:0047517 | 1,4-beta-D-xylan synthase activity | 29 | 21 | 6.21 | 8.22184875 | 6E-09 | MF |
| GO:0001872 | (1->3)-beta-D-glucan binding | 14 | 13 | 3 | 7.657577319 | 2.2E-08 | MF |
| GO:0016410 | N-acyltransferase activity | 103 | 36 | 22.07 | 7.602059991 | 2.5E-08 | MF |
| GO:0008569 | ATP-dependent microtubule motor activity, minus-end-directed | 16 | 14 | 3.43 | 7.48148606 | 3.3E-08 | MF |

BP: Biological process; CC: cellular component; MF: Molecular function.
